# Regulation of tumor growth by leukocyte-specific protein 1 in T cells

**DOI:** 10.1136/jitc-2020-001180

**Published:** 2020-10-05

**Authors:** Riri Kwon, Bong-Ki Hong, Kang-Gu Lee, Eunbyeol Choi, Laurent Sabbagh, Chul-Soo Cho, Naeun Lee, Wan-Uk Kim

**Affiliations:** 1 Center for Integrative Rheumatoid Transcriptomics and Dynamics, The Catholic University of Korea, Seoul, Republic of Korea; 2 Department of Biomedicine & Health Sciences, The Catholic University of Korea, Seoul, Republic of Korea; 3 Department of Microbiology, Infectiology, and Immunology, University of Montreal, Montreal, Quebec, Canada; 4 Division of Rheumatology, Department of Internal Medicine, The Catholic University of Korea, Seoul, Republic of Korea

**Keywords:** lymphocytes, tumor-infiltrating, T-lymphocytes, tumor microenvironment, melanoma, immunotherapy

## Abstract

**Background:**

Clinical efficacy of T cell-based cancer immunotherapy is limited by the lack of T cell infiltration in the tumor mass, especially in solid tumors. Our group demonstrated previously that leukocyte-specific protein 1 (LSP1), an intracellular signal regulator, negatively regulates T cell infiltration in inflamed tissues.

**Methods:**

To determine the immuno-regulatory effects of LSP1 in T cells on tumor progression, we investigated the growth of B16 melanoma in *Lsp1* knockout (KO) mice and T cell-specific *Lsp1* transgenic (Tg) mice. The immune cell subpopulation infiltrated into the tumor mass as well as the expression of interferon-gamma (IFN-γ) and tumor necrosis factor-alpha (TNF-α) in T cells was assessed by flow cytometry and/or immunohistochemistry. Chemotactic migration was assayed with *Lsp1* KO and *Lsp1* Tg T cells. Adoptive transfer of *Lsp1* KO or *Lsp1* Tg T cells was performed in B16 melanoma-challenged *Rag1* KO mice.

**Results:**

*Lsp1* KO mice showed decreased growth of B16 melanoma and increased infiltration of T cells in the tumor mass, which were completely reversed in T cell-specific *Lsp1* Tg mice. *Lsp1* KO CD8^+^ T cells also exhibited elevated migratory capacity in response to CXCL9 and CXCL10, whereas *Lsp1* Tg CD8^+^ T cells did the opposite. LSP1 expression was increased in tumor-infiltrating T cells and could be induced by T cell receptor activation. Intriguingly, gene expression profiling of *Lsp1* KO T cells suggested enhanced cytotoxicity. Indeed, expression of IFN-γ and TNF-α was increased in tumor-infiltrating CD4^+^ and CD8^+^ T cells of *Lsp1* KO mice, while it was markedly reduced in those of *Lsp1* Tg mice. Adoptive transfer of *Lsp1* KO T cells to *Rag1* KO mice was more effective in suppressing melanoma growth than transfer of *Lsp1* Tg T cells. Of note, when treated with antiprogrammed cell death protein 1 (PD-1) antibody, inhibition of melanoma growth was more pronounced in *Lsp1* KO mice than in *Lsp1-*sufficient mice, suggesting that *Lsp1* depletion additively increases the antitumor effects of anti-PD-1 antibody.

**Conclusions:**

LSP1 in T cells regulates the growth of B16 melanoma in mice, possibly by affecting migration and infiltration of T cells into the tumor and by modulating production of antitumor effector cytokines by CD8^+^ T cells. These findings provide evidence that LSP1 can be a target to improve the efficacy of T cell-based immunotherapy.

## Background

Immune contexture, which consists of the density, composition and functional status of tumor-infiltrating leukocytes (TILs), determines tumor progression and the efficacy of antitumor immunotherapy, including antibody (Ab)-based immunotherapy against programmed cell death protein 1 (PD-1).^1–3^ Several studies have suggested that a high density of T cells positively correlates with favorable prognosis and survival in patients with various cancers, including colorectal, non-small cell lung, hepatocellular, pancreatic and gastric cancers and melanoma.^1 2^ Therefore, adoptive cell transfer using antigen-activated T cells, particularly chimeric antigen receptor (CAR)-T cells, has emerged as one of the promising strategies to improve the efficacy of anticancer therapy.^4^ For example, CD19-targeted CAR-T cell therapy has shown remarkably high rates of remission in patients with hematological malignancies, including relapsed or refractory B-cell acute lymphoblastic leukemia and lymphoma.^4 5^

Despite its success in hematological malignancies, CAR-T cell therapy is not always efficacious and has shown rather disappointing results in some patients with solid tumors. One of the major hurdles of T cell-based cancer immunotherapies is insufficient trafficking of T cells into tumor masses.^4 6^ Despite the infusion of large amounts of T cells after ex vivo expansion, only a small portion of transferred T cells reaches inside the tumor tissues in clinical and preclinical studies.^7^ Although it remains unclear why trafficking, infiltration and penetration of T cells are insufficient, it may be primarily because solid tumors shape more fibrotic and less invasive environments through the activation of tumor-associated fibroblasts,^8^ ultimately constructing immunosuppressive tumor microenvironments (TME). Thus, to maximize the efficacy of T cell-based immunotherapy for solid tumors, it is essential to develop innovative ways for the successful delivery of immunocompetent T cells inside the tumor mass by destroying or detouring fibrotic and immunosuppressive TME.^8 9^

Leukocyte-specific protein 1 (LSP1) is an intracellular F-actin-binding protein that is mainly expressed in hematopoietic cells, such as T and B lymphocytes, neutrophils and macrophages.^10^ Previous studies have reported a negative regulatory role of LSP1 in leukocyte recruitment to inflamed sites.^10–12^ After peritoneal injection of thioglycolate or intra-articular injection of zymosan, infiltration of macrophages and neutrophils was found to be higher in inflamed tissues of *Lsp1* knockout (KO) mice than in those of wild-type (WT) mice.^11 12^ Recently, our group also demonstrated that loss of *Lsp1* promotes T cell migration into arthritic synovia and draining lymph nodes in mice with T cell-dependent chronic inflammation.^13^ Interestingly, several reports have suggested a possible link of *LSP1* to the pathogenesis of various cancers, including breast cancer,^14–16^ bladder cancer,^17^ dermatofibroma^18^ and hepatocellular carcinoma^19 20^ beyond its role in the migration of immune cells. For example, genetic variation in *LSP1* has been implicated in susceptibility, prognostic outcomes and as a diagnostic marker in diverse types of cancers.^14–19 21^ Moreover, a recent study showed that high LSP1 levels in glioblastoma serve as an independent predictive factor of unfavorable prognosis.^22^ However, it remains unclear whether LSP1 in T cells directly regulates tumor growth and how it contributes to the pathogenesis of cancers.

In this study, we postulated that *Lsp1* deficiency promotes the antitumor activity of T cells by inducing cell migration and invasion into the tumor mass. We demonstrated that *Lsp1* deficiency in T cells suppresses the growth of B16 melanoma in mice, which seems to be mediated by increased infiltration of CD8^+^ T cells into tumor sites and by enhanced production of interferon-gamma (IFN-γ) and tumor necrosis factor-alpha (TNF-α), antitumor effector cytokines, by T cells. In contrast, *Lsp1*-overexpressing T cells show the opposite results. Notably, *Lsp1* KO further potentiates the suppressive effect of anti-PD-1 Ab on melanoma growth. Together, these results suggest that LSP1 depletion in T cells can be an effective strategy to overcome the current limitations of T cell-based immunotherapy and to improve the efficacy of anti-PD-1 Ab for solid tumors.

## Materials and methods

### Animals

Mice genetically deficient in the *Lsp1* gene (*Lsp1* KO) on the C57BL/6 background were kindly provided by Dr Laurent Sabbagh (University of Montreal, Montreal, Quebec, Canada).^23^ For the generation of *Lsp1* transgenic (*Lsp1* Tg) mice in which the *Lsp1* gene was specifically overexpressed in T cells, mouse *Lsp1* cDNA was cloned into a lymphocyte-specific expression cassette, including the human CD2 promoter. The construct was injected directly into the pronucleus of fertilized eggs and the transgenic founder was isolated by PCR of genomic DNA. To detect the *Lsp1* transgene in *Lsp1* Tg mice, genomic DNA was extracted from tails of WT and *Lsp1* Tg mice, and then PCR analysis of the *Lsp1* transgene was performed using the following primer sequences: forward, 5’-GGACTCCACCAGTCTCACTTCAG-3’ and reverse, 5’-CAGTTCAGAGGACTTCAGGCTGAT-3’. G protein signaling 7 gene (*Rgs7*) was used as an internal control with the primers 5’-CAACCACTTACAAGAGACCCGTA-3’ and 5’-GAGCCCTTAGAAATAACGTTCACC-3’.

For the adoptive transfer experiments using T cells, *Rag1* KO mice were obtained from Jackson Laboratory (Bar Harbor, Maine, USA). All strains were in the C57BL/6 background, and age-matched and sex-matched WT C57BL/6 mice were used as a control.

### Induction of B16 melanoma in mice

The B16BL6 melanoma cell line (hereafter termed ‘B16 melanoma’) was purchased from the Korean Cell Line Bank (Seoul, Korea). The Lewis lung carcinoma (LLC) cell line was obtained from the American Type Culture Collection (Manassas, Virginia, USA). The MC38 colon adenocarcinoma cell line was kindly provided by Dr Tai-Gyu Kim (The Catholic University of Korea, Seoul, Korea). All cell lines were cultured in Dulbecco’s Modified Eagle Medium (Welgene, Gyeongsan, Korea) supplemented with 10% heat-inactivated fetal bovine serum (FBS), 100 U/mL penicillin, 100 μg/mL streptomycin and 0.25 μg/mL Fungizone (Gibco; Thermo Fisher Scientific, Waltham, Massachusetts, USA). All cell lines used in this study were negative for *Mycoplasma*, when tested using an e-Myco Mycoplasma PCR Detection Kit (iNtRON Biotec, Seongnam, Korea). After being resuspended in phosphate buffered saline (PBS), 5×10^5^ B16 melanoma cells, 1×10^5^ MC38 cells or 2.5×10^5^ LLC cells were injected subcutaneously into the right flank of mice aged 8–12 weeks. Tumor volumes were measured every 2–3 days with a caliper and calculated according to the following formula: V(mm^3^)=D×d^2^×0.52, where D (mm) and d (mm) are the largest and smallest perpendicular tumor diameters, respectively.

### Isolation of TILs

For isolation of TILs, the mice were sacrificed when the average WT tumor volume reached 500 or 700 mm^3^. After euthanizing mice, primary tumors were excised and dissociated by mechanical force. Tumor cell suspensions were obtained after filtration with a 70 μm cell strainer. TILs were isolated using Ficoll-Hypaque (GE Healthcare, Chicago, Illinois, USA) density gradient centrifugation. The single-cell suspensions were washed in PBS and then subjected to flow cytometry analysis or in vitro re-stimulation for intracellular cytokine staining.

### Flow cytometry

Single-cell suspensions were prepared from the tumor tissues and spleens of WT, *Lsp1* KO and *Lsp1* Tg mice after tumor inoculation. Surface staining was performed for 30 min at 4°C with the following fluorochrome-labeled antimouse Abs: CD45 (30-F11, BD Pharmingen, Franklin Lakes, New Jersey, USA), CD3 (145-2 C11, Invitrogen, Carlsbad, California, USA), CD4 (GK1.5, Biolegend, San Diego, California, USA), CD8 (53–6.7, Biolegend), CD19 (eBio 1D3, Invitrogen), NK1.1 (PK136, Biolegend), CD11b (M1/70, Biolegend), Ly-6C (HK1.4, Invitrogen), Ly-6G (1A8, Biolegend), F4/80 (BM8, Invitrogen), CD206 (C068C2, Biolegend), major histocompatibility complex (MHC) class II (M5/114.15.2, Biolegend) and CXCR3 (S18001A, Biolegend). To detect cytokine production, splenocytes and TILs were re-stimulated in vitro with phorbol-12-myristate-13-acetate (50 ng/mL, Sigma, St. Louis, MO) plus ionomycin (500 ng/mL, Sigma) for 4 hours in the presence of GolgiPlug (BD Bioscience). After surface staining, cells were fixed, permeabilized and stained with the following fluorochrome-labeled Abs: interleukin (IL)-2 (JES6-5H4, Biolegend), TNF-α (MP6-XT22, Biolegend), IFN-γ (XMG1.2, BD Biosciences) and Foxp3 (NRRF-30, Invitrogen) for 1 hour at 4°C. Intracellular expression of LSP1 in T cells also was detected by flow cytometry. In brief, cells were stained with surface markers for 30 min at 4°C. After surface staining, cells were fixed, permeabilized and incubated with rabbit anti-LSP1 Ab (Cell Signaling Technology, Danvers, Massachusetts, USA) or recombinant rabbit IgG (Abcam, Cambridge, UK) for 1 hour and then stained with a fluorescein isothiocyanate (FITC)-conjugated secondary Ab (Invitrogen) for 30 min to detect LSP1 expression in B16-challenged TILs, or they were stained with FITC-conjugated mouse anti-LSP1 Ab (mouse IgG_1_; BD Biosciences) for 1 hour to detect LSP1 expression in in vitro stimulated T cells. FITC-conjugated mouse IgG_1_ (Santa Cruz Biotechnology, Dallas, Texas, USA) was used as an isotype control. Cells were resuspended in fluorescence-activated cell sorting (FACS) buffer and acquired through FACS Canto II (BD Biosciences) or LSR Fortessa (BD Biosciences) with DIVA software. All data were analyzed using FlowJo software (FlowJo, Franklin Lakes).

### Immunohistochemistry

For immunohistochemical staining, 7 μm sections of Optimal Cutting Temperature (OCT)-embedded tumor tissues were fixed with cold acetone for 10 min at −20°C, endogenous peroxidase was quenched by incubating the sections in 0.3% H_2_O_2_ for 30 min at room temperature and then tissues were blocked with 10% normal donkey serum for 1 hour at room temperature. Tissue sections were incubated with rat antimouse CD4 (1:1000, Biolegend) or rat antimouse CD8 (1:1000, Biolegend) Ab overnight at 4°C. Each slide was washed three times with PBS and detected using an antirat secondary Ab (1:100, Vector Laboratories, Burlingame, California, USA) with the VECTASTAIN Elite ABC HRP kit (Vector Laboratories). Positive cells were detected using 3,3′-diaminobenzidine tetrahydrochloride (Vector Laboratories) and counterstained with hematoxylin. Images were obtained using a Pannoramic MIDI slide scanner (3DHISTECH). Positive cells were counted manually in six fields per slide, with two different slides per mouse.

### Quantitative real-time PCR

Total RNA was isolated from T cells of mouse spleen using the RNeasy Mini kit (Qiagen, Hilden, Germany), according to the manufacturer’s instructions. For real-time quantitative PCR (qPCR), RNA was reverse transcribed to cDNA using RevertAid Reverse Transcriptase (Thermo Fisher Scientific), and qPCR was performed on the CFX96 real-time PCR system using SYBR Green PCR premix (Bio-Rad, Hercules, California, USA). The primer sequences used for detection of *Lsp1* mRNA expression were 5’-CCAGCCCTTTGGCCTTAGAA-3’ and 5’-TGGAAATGGGCAAGGTTGGT-3’. All samples were normalized to *Gapdh* expression detected using the primers 5’-AGGTCGGTGTGAACGGATTTG-3’ and 5’-TGTAGACCATGTAGTTGAGGTCA-3’ and relative fold-change was calculated using the 2^−ΔΔCt^ method.

### Western blot analysis of LSP1 and p-Akt

Expression of LSP1 and phosphorylated Akt (p-Akt) in T cells was detected by western blot analysis. Briefly, stimulated T cells were lysed in a lysis buffer, and final protein concentrations were determined using a Bradford protein assay (Bio-Rad). Total protein was separated on 12% SDS-PAGE gels and transferred to a polyvinylidene fluoride membrane by electroblotting. The membranes were incubated with Abs against LSP1 (1:1000), p-Akt (1:500), Akt (1:1000, all from Cell Signaling Technology) or β-tubulin (1:1000, Abcam), followed by horseradish peroxidase-conjugated antirabbit IgG (Thermo Fisher Scientific). The membranes were visualized using an enhanced chemiluminescent detection system (Thermo Fisher Scientific).

### T cell migration assay

Chemotaxis of WT, *Lsp1* KO and *Lsp1* Tg CD8^+^ T cells was performed in 24-well plates with 5 μm pore size Transwell inserts (Corning, Corning, New York, USA). Murine CXCL9 and CXCL10 (R&D Systems, Minneapolis, Minnesota, USA) were diluted to the indicated concentrations in migration medium (0.1% FBS in RPMI1640) and placed in the lower chamber. A half million CD8^+^ T cells were loaded into the upper chamber in migration medium. After 4 hours of incubation at 37°C, the cells that had migrated to the lower chamber were counted using a hemocytometer. In some experiments, WT and *Lsp1* KO CD4^+^ T cells were co-cultured with carboxyfluorescein succinimidyl ester (CFSE, 1 μM, Thermo Fisher Scientific)-labeled WT CD8^+^ T cells in the absence or presence of anti-CD3 Ab. After 3 days, cells were harvested and resuspended in migration medium and loaded in the upper chamber after CXCL9 or CXCL10 were added in the bottom chamber. After 4 hours of incubation, CFSE-labeled CD8^+^ T cells that had migrated to the lower chamber were calculated by flow cytometry.

### T cell culture

Splenic T cells of WT mice were cultured to examine the major stimuli and their signaling pathways to induce LSP1 expression. Briefly, T cells were isolated from the spleens and prepared as single-cell suspensions. CD4^+^ T cells or CD8^+^ T cells were purified by magnetic separation using anti-CD4 beads or anti-CD8 beads (Miltenyi Biotec, Bergisch Gladbach, Germany) according to the manufacturer’s instructions. Purified CD4^+^ T cells or CD8^+^ T cells were stimulated with recombinant IFN-γ (10 ng/mL, R&D Systems), transforming growth factor-β (TGF-β, 2 ng/mL, R&D Systems), IL-10 (10 ng/mL, R&D Systems) or antimouse CD3ε Ab (1 µg/mL, 145-2 C11, Invitrogen) plus antimouse CD28 Ab (1 µg/mL, 37.51, Invitrogen) in complete media for 72 hours. In some experiments, ciclosporin A (Sigma), tacrolimus (FK506, Sigma) and rapamycin (Sigma) were treated to the T cells stimulated with anti-CD3/anti-CD28 Abs for 72 hours to determine whether the calcineurin pathway is involved in LSP1 expression. The cultured cells were harvested and stained to detect intracellular LSP1 expression by flow cytometry and/or western blot analysis.

### T cell proliferation and apoptosis assay

T cell proliferation was assessed by flow cytometry analysis of CFSE-stained cells according to the manufacturer’s instructions. Briefly, isolated CD4^+^ or CD8^+^ T cells were resuspended in PBS at a density of 1×10^7^ cells/mL and incubated with 1 μM of CFSE (Invitrogen) for 10 min at room temperature in the dark. Stained cells were quenched using FBS for 10 min on ice. The cells were washed twice and resuspended in complete RPMI 1640 medium and then analyzed by flow cytometry. Apoptosis was measured using the FITC-Annexin V Apoptosis Detection Kit (BD Bioscience) according to the manufacturer’s protocol. In brief, cultured T cells were harvested, washed with PBS and resuspended in annexin V-binding buffer. The cells were gently mixed with FITC-annexin V and propidium iodide (PI) and then incubated for 15 min at room temperature in the dark. Subsequently, annexin V^+^ and/or PI^+^ cells were analyzed by flow cytometry.

### Microarray and gene set enrichment analysis

Total RNA was isolated from splenic T cells of *Lsp1* KO and WT mice, which were stimulated with anti-CD3/anti-CD28 Abs for 6 hours. The RNA was reverse-transcribed, amplified according to standard Agilent protocols, and then hybridized to an array chip (SurePrint G3 Mouse GE 8×60K Microarray, Agilent) containing 62 976 probes for 24 241 annotated genes (GSE75123). Briefly, after normalization, the log2 fold-change values and p values of each gene were calculated as previously described.^13^ The cut-off values of differentially expressed genes (DEGs) in *Lsp1* KO T cells were as follows: |fold-change values| > |the fold-change values of 2.5th and 97.5th percentile of the empirical null distribution| and p values <0.05. Functional enrichment analysis of DEGs was performed using DAVID Bioinformatics Resources 6.8 (https://david.ncifcrf.gov/). The GOBP terms of leukocyte related were first selected based on their titles and definitions. Gene set enrichment analysis (GSEA) was performed by clusterProfiler (R package, V.3.4.6)^24^ and GSEA plots were generated with enrichrplot (R package).

### Adoptive transfer of T cells to Rag1 KO mice

*Rag1* KO mice were inoculated subcutaneously with 1×10^5^ B16 melanoma cells in the right flank. Next day, T cells were isolated from spleens of non-tumor-bearing *Lsp1* KO and *Lsp1* Tg mice by magnetic separation using a Pan T cell isolation kit (Miltenyi Biotec) according to the manufacturer’s instructions. PBS as a vehicle or 1×10^7^ T cells from *Lsp1* KO or *Lsp1* Tg mice were injected intravenously into B16 melanoma-bearing *Rag1* KO mice. Tumor growth was recorded every other day.

### Combination immunotherapy with anti-PD-1 blockade

For anti-PD-1 blockade therapy, 5×10^5^ B16 melanoma cells were inoculated subcutaneously into the right flank of WT and *Lsp1* KO mice. On days 3, 6, 9 and 12 after tumor inoculation, the tumor-bearing mice were treated intraperitoneally with 10 mg/kg anti-PD-1 Ab (RMP1-14, Bio X cell, Lebanon, New Hampshire, USA) or matched rat IgG2a isotype control (2A3, Bio X cell). Tumor growth was monitored every other day.

### Statistical analysis

Statistical analysis was conducted using GraphPad Prism software. The unpaired t*-*test was used for statistical evaluations as indicated in each experiment, while two-way analysis of variance analysis was performed to determine the significance of tumor growth. Data are shown as the mean±SD. P values <0.05 were considered to be statistically significant.

## Results

### LSP1 deficiency restrains tumor growth while promoting T cell infiltration in tumors

**Figure 1:**
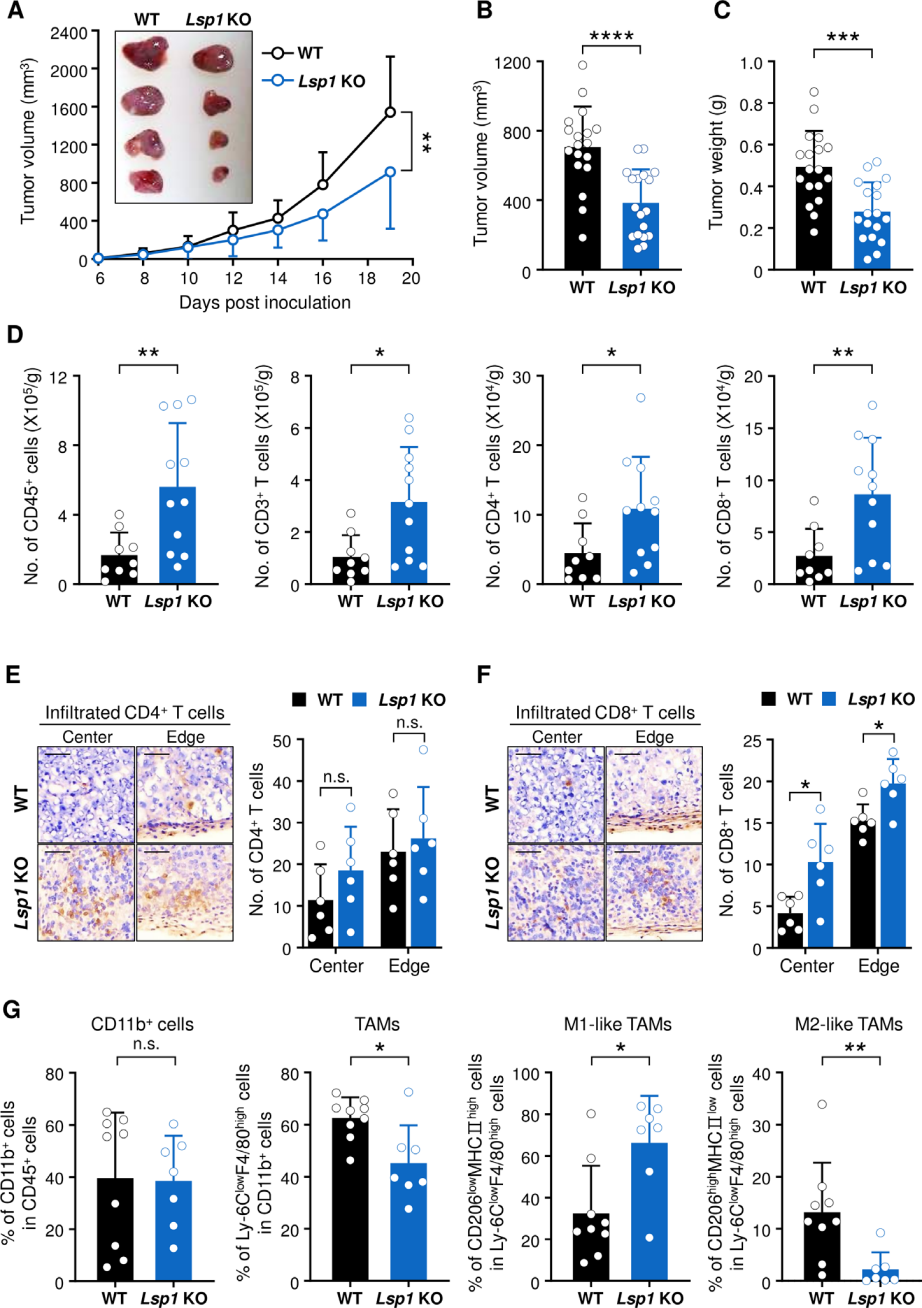
Effect of leukocyte-specific protein 1 (*Lsp1*) deficiency on melanoma growth and T cell infiltration in tumors. (A) Tumor growth in wild-type (WT) (n=11) and *Lsp1* knockout (KO) mice (n=9). Mice were subcutaneously injected with B16 melanoma cells and tumor growth was measured at the indicated time points. (B and C) Tumor volume and weight in WT and *Lsp1* KO mice (n=18 per group). When the average volume of tumors in WT mice reached approximately 700 mm^3^, tumor weight was assessed in the two groups of mice. (D) The number (No.) of tumor-infiltrating leukocytes (TILs), in tumors from B16 melanoma-challenged WT (n=9) and *Lsp1* KO mice (n=11), which was assessed by flow cytometry at the same time, as described in (B and C). The gating strategy for flow cytometry analysis is shown in online supplemental figure 2A. (E and F) Immunohistochemical staining of CD4^+^ or CD8^+^ T cells in frozen sections of melanoma obtained from WT and *Lsp1* KO (n=3 per group) mice. Representative images of CD4^+^ T cells (E, left panel) and CD8^+^ T cells (F, left panel) infiltrated into the center or edge of the tumor are shown in brown. The sections were counterstained with hematoxylin. Scale bar indicates 50 μm for all images. The mean number of infiltrating CD4^+^ (E, right panel) or CD8^+^ (F, right panel) T cells was calculated from two slides per tumor tissue and five to six fields per slide. (G) Comparison of the frequencies of CD11b^+^ cells in CD45^+^ leukocytes, Ly-6C^low^F4/80^high^ cells (tumor-associated macrophages (TAMs)) in CD11b^+^ cells, and CD206^low^MHCII^high^ (M1-like TAMs) or CD206^high^MHCII^low^ cells (M2-like TAMs) in Ly-6C^low^F4/80^high^ cells between WT (n=9) and *Lsp1* KO mice (n=7). Representative zebra plots and the gating strategy for flow cytometry analysis are presented in online supplemental figure 2D. Data are the mean±SD of at least two independent experiments. The circle in the bar graphs represents the individual value. P values were determined by two-way analysis of variance analysis (A) or unpaired two-tailed t-test (B–G). n.s., not significant. *P<0.05; **p<0.01; ***p<0.001; ****p<0.0001.

The role of LSP1 for tumor immunity remains to be defined. To address this issue, we subcutaneously inoculated syngeneic B16 melanoma cells into WT and *Lsp1* KO mice and then observed tumor growth over 3 weeks. We found that *Lsp1* KO mice had significant reductions in tumor growth as compared with WT mice (figure 1A). Moreover, tumor volume and weight in *Lsp1* KO mice were lower than in WT mice as determined on day 14 after tumor inoculation (figure 1B, C). To explore whether the inhibitory effect of *Lsp1* deficiency on tumor growth is reproduced in other types of solid tumor, we assessed the growth of MC38 colon cancer in WT and *Lsp1* KO mice. Similar to the results obtained in the B16 melanoma model, the growth of MC38 colon cancer cells was significantly diminished in *Lsp1* KO mice (online supplemental figure 1A). However, there was no difference in the growth of LLC between WT and *Lsp1* KO mice (online supplemental figure 1B).

10.1136/jitc-2020-001180.supp1 Supplementary data

To characterize the effects of *Lsp1* deficiency on immune contexture in the TME, TILs in tumor-bearing WT and *Lsp1* KO mice were first analyzed using flow cytometry when the average tumor volume in WT mice reached approximately 700 mm^3^. The results showed that tumors derived from *Lsp1* KO mice had a greater number of infiltrated CD45^+^, CD3^+^, CD4^+^ and CD8^+^ T cells than those from WT mice (figure 1D) and that the frequencies of those cells were not different between WT and *Lsp1* KO mice (online supplemental figure 2A, B). These results imply that reduced melanoma growth in *Lsp1* KO mice may be related to the increased numbers of T cells rather than composition. A growing body of evidence suggests that the spatial distribution of TILs, specifically whether the cells are located at the center or invasive margin of a tumor, as well as immune heterogeneity of TILs, critically determines the responsiveness to antitumor immunotherapy.^1 25 26^ In this melanoma model, infiltration of CD8^+^ T cells, but not that of CD4^+^ T cells, was significantly higher in both the center and edge regions of the tumors in *Lsp1*-deficient mice than in WT mice, as assessed by immunohistochemical staining (figure 1E, F), indicating that *Lsp1* deficiency promotes CD8^+^ T cell infiltration into the tumor center.

In addition to T cells, other immune cell populations contribute to the TME. We found that there were no differences in the frequencies of intratumoral NK1.1^+^ natural killer (NK) cells and CD19^+^ B cells (online supplemental figure 2C), even though the absolute numbers of TILs were higher in the tumors derived from *Lsp1* KO mice. The frequency of regulatory T cells (T_reg_ cells: Foxp3^+^ CD4^+^ T cells), a representative subset of immunosuppressors in the tumor milieu, also showed no difference (online supplemental figure 2C). Among TILs, the myeloid cell population is another substantial component of the TME that regulates tumor growth.^1 8^ Interestingly, the frequency of CD11b^+^Ly6C^low^F4/80^high^ tumor-associated macrophages (TAMs) was also significantly decreased in the tumors of *Lsp1* KO mice compared with those of WT mice, although the frequency of CD11b^+^ myeloid cells was similar between *Lsp1* KO and WT mice (online supplemental figure 2D and figure 1G). Of note, among TAMs, there was a significant increase of pro-inflammatory M1-like (CD206^low^ MHCII^high^) TAMs and a substantial decrease of anti-inflammatory M2-like (CD206^high^ MHCII^low^) TAMs in the tumors of *Lsp1* KO mice (online supplemental figure 2D and figure 1G).

Collectively, these results demonstrate that *Lsp1*-deficient mice establish a more favorable antitumor immune milieu by enhancing infiltration of pro-inflammatory M1-like rather than M2-like TAMs as well as that of CD8^+^ T cells.

### LSP1 overexpression in T cells promotes melanoma growth while suppressing T cell infiltration in tumors

**Figure 2:**
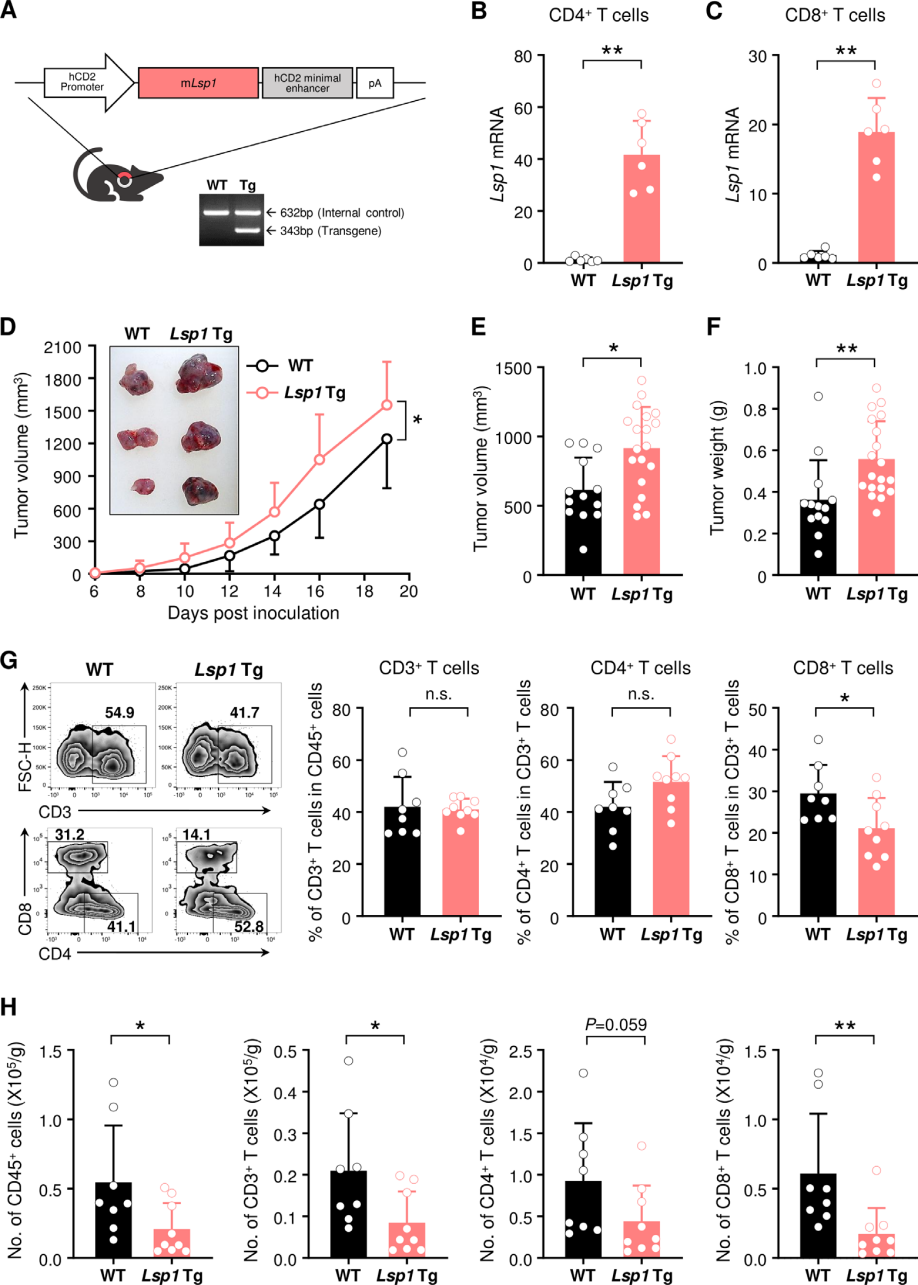
Increase in growth of melanoma and decrease in infiltration of T cells by T cell-specific leukocyte-specific protein 1 (*Lsp1*) overexpression. (A) Generation of T cell-specific *Lsp1-*overexpressing mice. Diagram shows the structure of the *Lsp1* transgeni*c* (Tg) construct containing a hCD2 promoter-*Lsp1* transgene (top panel). PCR analysis of the *Lsp1* transgene and an internal control gene (G protein signaling 7; *Rgs7*) was performed with genomic DNA isolated from wild-type (WT) and *Lsp1* Tg mice (bottom panel). (B and C) Quantitative real-time PCR analysis of *Lsp1* mRNA expression levels in splenic CD4^+^ T cells and CD8^+^ T cells of WT and *Lsp1* Tg mice (n=6 per group). *Gapdh* was used as an internal control. Fold inductions were calculated using the 2^-ΔΔCt^ method. (D) Tumor growth in WT (n=12) and *Lsp1* Tg mice (n=11). Mice were subcutaneously injected with B16 melanoma cells and tumor growth was measured at the indicated time points. (E and F) Tumor volume and weight in WT (n=13) and *Lsp1* Tg mice (n=19). When the average volume of tumors in WT mice reached approximately 500 mm^3^, tumor weight was assessed in the two groups of mice. (G and H) Flow cytometry analysis of T cells infiltrated in B16 melanoma. The cells were isolated from the tumors of WT (n=8) and *Lsp1* Tg mice (n=9) at the same time as described in (E and F). The frequencies of CD3^+^ T cells in CD45^+^ leukocytes and those of CD4^+^ or CD8^+^ T cells in CD3^+^ T cells are shown in (G) as representative zebra plots and bar graphs. The number (No.) of T cells is presented in (H). Data are the mean±SD of at least two independent experiments. The circle in the bar graphs indicates the individual value. P values were determined by two-way analysis of variance analysis (D) or unpaired two-tailed t-test (B, C and E–H). n.s., not significant. *P<0.05; **p<0.01.

To extend our understanding of the T cell-specific effects of LSP1 during tumor development, we generated transgenic (Tg) mice that specifically overexpress *Lsp1* in T cells using CD2 promoter, as described in ‘*Materials and methods*’ section (figure 2A). We first confirmed that CD4^+^ and CD8^+^ T cells in *Lsp1* Tg mice expressed much higher levels of *Lsp1* mRNA than those in WT mice (figure 2B, C). WT and *Lsp1* Tg mice were subcutaneously challenged with B16 melanoma cells, as shown in figure 1. In sharp contrast with *Lsp1* KO mice, *Lsp1* Tg mice showed substantial acceleration of tumor growth over a period of 3 weeks in comparison to WT mice (figure 2D). At day 14 after tumor inoculation, the volume and weight of the tumors derived from *Lsp1* Tg mice were also significantly higher than those of WT mice (figure 2E, F).

Since CD2 promoter drives early expression of the transgene in the double negative stage of thymocytes, it is conceivable that high LSP1 expression in CD2^+^ T cells would affect T cell development in the thymus. Here, we found no differences in the number of total thymocytes and the frequencies of cells in thymus developmental stages from double negative cells to single positive cells between WT and *Lsp1* Tg mice, as determined by flow cytometry (online supplemental figure 3A). Additionally, no differences were observed in the peripheral T cell numbers and the ratio of CD4^+^ or CD8^+^ T cell population in blood and spleens of WT versus *Lsp1* Tg mice (online supplemental figure 3B, C). Together, these data suggest that CD2-driven *Lsp1* overexpression does not affect T cell education and homeostasis. In support of this notion, it has been demonstrated that *Lsp1* deficiency does not affect T cell development and homeostasis in the central and peripheral tissues.^11^

To evaluate the immune cell population in the TME of *Lsp1* Tg mice, tumors were collected when tumor volume derived from WT mice reached approximately 500 mm^3^ and then the number and frequency of TILs were analyzed using flow cytometry (figure 2G). The results showed that the frequencies of tumor-infiltrating CD3^+^ and CD4^+^ T cells were similar between WT and *Lsp1* Tg mice. However, in contrast to the results in *Lsp1* KO mice, the frequency of CD8^+^ T cells was reduced in *Lsp1* Tg as compared with WT mice (figure 2G). The absolute number of intratumoral CD45^+^, CD3^+^ and, especially, CD8^+^ T cells was also significantly lower in *Lsp1* Tg mice than in WT mice (figure 2H). As expected, since *Lsp1* overexpression is specific to T cells, no difference was found in the frequency of intratumoral NK1.1^+^ NK cells, CD19^+^ B cells, CD11b^+^ myeloid cells and even TAMs between the two types of mice (online supplemental figure 4). Collectively, these results show that specific overexpression of *Lsp1* in T cells enhances B16 melanoma growth, which is associated with a decrease in the number and frequency of TILs, particularly CD8^+^ T cells.

The decreased infiltration of *Lsp1* Tg CD8^+^ T cells could be due to the reduction of T cell survival and proliferation. To test this possibility, we checked the proliferation and survival of B16-challenged *Lsp1* Tg versus WT T cells in the presence or absence of anti-CD3/anti-CD28 Abs using flow cytometry. As shown in online supplemental figure 5, no differences were found in the frequencies of CFSE-diluted cells or annexin V^+^ and/or PI^+^ cells in CD4^+^ and CD8^+^ T cells between the two groups of mice, suggesting that other mechanism(s) than cell proliferation and survival are responsible for the decrease of TILs in the melanoma of *Lsp1* Tg mice.

### LSP1 negatively regulates CD8^+^ T cell migration

**Figure 3:**
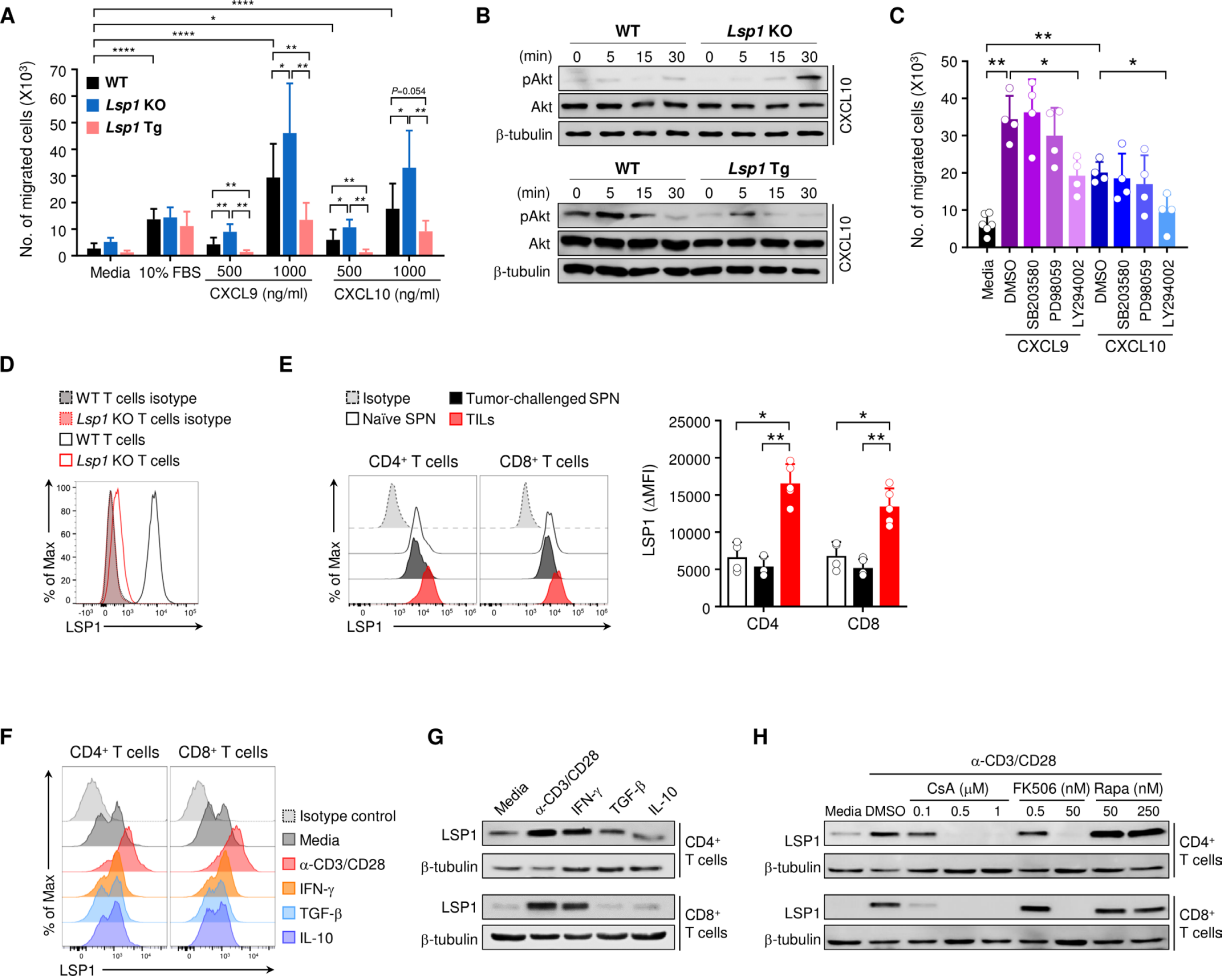
Leukocyte-specific protein 1 (LSP1) expression in T cells and its role in T cell migration. (A) Chemotactic migration of CD8^+^ T cells of wild-type (WT) (n=13), *Lsp1* knockout (KO) (n=5) and *Lsp1* Tg mice (n=9). (B) Western blot analysis for phosphorylated Akt (p-Akt) expression. WT, *Lsp1* KO and *Lsp1* Tg CD8^+^ T cells were treated with CXCL10 (1000 ng/mL) for the indicated time. p-Akt and Akt expression in the cells was determined by western blot analysis. (C) Decreases in WT CD8^+^ T cell migration by a specific inhibitor of Akt. WT CD8^+^ T cells were treated with p38 MAPK inhibitor (SB203580, 10 μM), ERK inhibitor (PD98059, 2 μM) or PI3K/Akt inhibitor (LY294002, 20 μM) for 30 min. Cell migration induced by CXCL9 (1000 ng/mL) or CXCL10 (1000 ng/mL) was assayed using a hemocytometer. (D) Specificity of antimouse LSP1 antibody (Ab) used in flow cytometry. (E) LSP1 expression levels in tumor-infiltrating CD4^+^ T cells and CD8^+^ T cells. Fifteen days after inoculation of B16 melanoma in WT mice (n=5), tumor-infiltrating leukocytes (TILs) were isolated from tumor tissues. Spleen cells (SPN) of WT mice with tumor inoculation (tumor-challenged SPN, n=5) and those without tumor inoculation (naïve SPN, n=4) were used as controls. Intracellular LSP1 expression levels were assessed using flow cytometry. (F and G) Increase in LSP1 expression in T cells by stimulation with anti-CD3/anti-CD28 Abs and interferon-gamma (IFN-γ). WT splenic T cells were stimulated with IFN-γ (10 ng/mL), transforming growth factor-β (TGF-β) (2 ng/mL), interleukin (IL)-10 (10 ng/mL) or anti-CD3 plus anti-CD28 Abs (α-CD3/CD28, 1 µg/mL) for 72 hours. Intracellular LSP1 expression levels were measured by flow cytometry (F) and western blot analysis (G). (H) Suppression of LSP1 expression by calcineurin inhibitors. WT splenic T cells were stimulated with anti-CD3/anti-CD28 Abs (α-CD3/CD28, 1 µg/mL) in the absence or presence of ciclosporin A, tacrolimus or rapamycin at the indicated concentrations for 72 hours. LSP1 expression in the cells were measured by western blot analysis. Data in (A to H) are representative of at least three independent experiments or the mean±SD. P values were determined by unpaired two-tailed t-test. *P<0.05; **p<0.01; ***p<0.001; ****p<0.0001.

Previously, we demonstrated that *Lsp1* deficiency directly increases CD4^+^ T cell migration in response to stromal cell-derived factor-1, a major chemokine that is known to be involved in CD4^+^ T cell migration under arthritis-associated conditions.^13^ In this study, we tested whether LSP1 affects CD8^+^ T cell migration under tumor-associated conditions. It is widely accepted that the CXCR3-CXCL9/CXCL10 axis has a crucial role in driving the trafficking of CD8^+^ T cells to tumor sites.^27^ Moreover, activation of that axis promotes the interaction between tumor-specific T cells and dendritic cells in the TME during anti-PD-1 therapy.^28^ We, therefore, investigated whether LSP1 controls CD8^+^ T cell migration in response to CXCL9 and CXCL10. We found that *Lsp1*-deficient CD8^+^ T cells showed a greater chemotactic response to CXCL9 and CXCL10 than WT CD8^+^ T cells (figure 3A). By contrast, *Lsp1*-overexpressing CD8^+^ T cells displayed diminished chemotactic migration compared with WT and *Lsp1*-deficient CD8^+^ T cells (figure 3A), demonstrating that LSP1 negatively regulates the migration of CD8^+^ T cells. However, migration of CD8^+^ T cells stimulated with 10% FBS did not differ among the three types of CD8^+^ T cells (figure 3A), suggesting that LSP1 regulation of CD8^+^ T cell migration is specific to CXCL9 and CXCL10.

To mechanistically understand how T cell migration is modulated by LSP1 expression, we first examined the expression level of CXCR3, a specific receptor of CXCL9 and CXCL10, on T cells of WT, *Lsp1* KO and *Lsp1* Tg mice. As shown in online supplemental figure 6A and B, no differences in CXCR3 expression levels were observed among the three genotypes. Additionally, our microarray data showed that there was a paucity of chemokine receptor-related genes in the DEGs (online supplemental figure 6C), implying that expression of chemokine receptors, particularly with CXCR3, is not relevant to LSP1 control of T cell migration. Earlier studies demonstrated that CXCL9/10-CXCR3 axis transmits its signals through the Akt.^29^ Therefore, we next investigated to determine if Akt is a downstream target of LSP1 for T cell migration. As shown in figure 3B, the expression level of p-Akt was reduced in *Lsp1* Tg CD8^+^ T cells on CXCL10 stimulation, as determined by western blot analysis. Conversely, CXCL10-triggered-p-Akt expression was higher in CD8^+^ T cells of *Lsp1* KO mice than in those of WT mice (figure 3B), indicating that LSP1 is a negative regulator of Akt activation. CXCL9-stimulated *Lsp1* Tg CD8^+^ T cells showed similar results (data not shown). Moreover, the CXCL9/10-induced increase in CD8^+^ T cell migration was almost completely abrogated by the Akt inhibitor LY294002, but not by the Erk inhibitor PD98059 or p38 inhibitor SB203580 (figure 3C). Overall, these results suggest that LSP1 inhibits CXCL9/10-induced T cell migration by regulating the extent of Akt phosphorylation.

To further demonstrate the pathological relevance of LSP1 in T cells to tumor conditions, we investigated LSP1 expression levels in TILs of B16 melanoma by flow cytometry. Specificity of the anti-LSP1 Ab was validated, as shown in figure 3D. As shown in figure 3E, CD4^+^ T cells infiltrated into B16 melanoma tissue, but not splenic CD4^+^ T cells in the same mice, exhibited substantially higher LSP1 expression than splenic T cells of non-tumor-bearing mice. CD8^+^ T cells infiltrated into B16 melanoma tissue showed similar results, demonstrating that high levels of LSP1 in T cells are possibly induced by B16 melanoma. To better understand how the upregulation of LSP1 expression occurs in tumor-infiltrating T cells in vivo, we investigated which kinds of tumor-associated stimuli can induce LSP1 expression. As shown in figure 3F, stimulation of T cells with anti-CD3/anti-CD28 Abs or IFN-γ, which is known as a pro-inflammatory cytokine abundantly produced in the TME,^30^ strongly increased LSP1 expression in both CD4^+^ and CD8^+^ T cells. The increase in LSP1 expression by T cell receptor (TCR) activation and IFN-γ was confirmed by western blot analysis (figure 3G). By contrast, TGF-β and IL-10, anti-inflammatory cytokines derived from the TME,^8 31^ failed to upregulate LSP1 expression (figure 3F, G). Ciclosporin A and tacrolimus (FK506), specific calcineurin inhibitors, markedly suppressed anti-CD3/anti-CD28 Abs-induced increase in LSP1 expression, while rapamycin failed to do so, indicating that LSP1 induction by TCR activation is calcineurin-dependent (figure 3H). Given that LSP1 in T cells negatively regulates T cell migration, these results suggest that B16 melanoma can evade the antitumor activity of host T cells by upregulating LSP1 expression in T cells within the TME.

### Lsp1-deficient T cells show increased cytotoxicity

**Figure 4:**
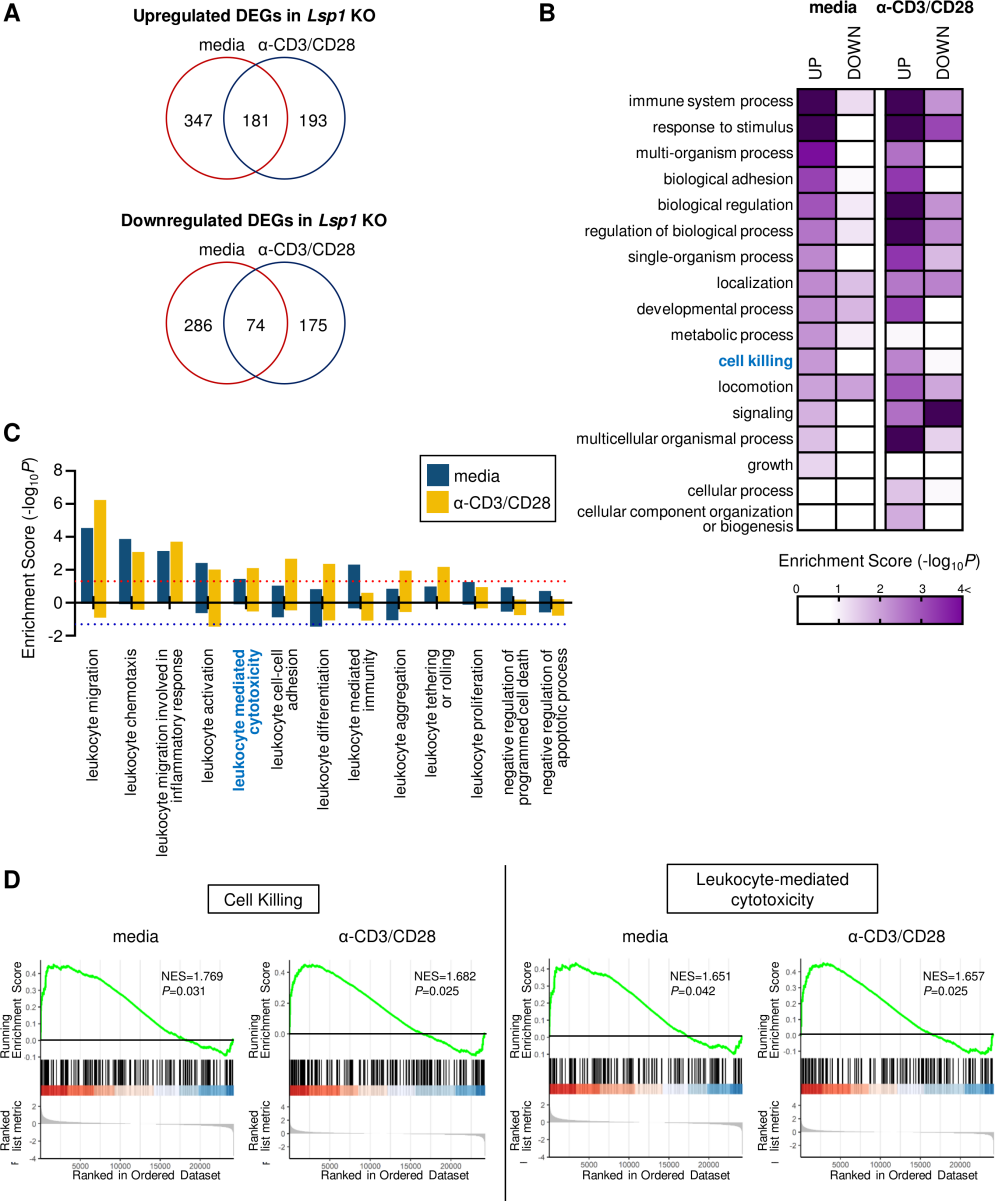
Increased cytotoxicity-related biological processes in leukocyte-specific protein 1 (*Lsp1*) knockout (KO) T cells. (A) Venn diagram depicting the overlap between the differentially expressed genes (DEGs) in *Lsp1* KO T cells compared with wild-type (WT) T cells stimulated media alone and those with anti-CD3/anti-CD28 antibodies (Abs) (α-CD3/CD28) for 6 hours. The RNA was reverse-transcribed, amplified and then hybridized to microarray chips as described in ‘*Materials and methods*’ section (GSE75123). (B) Heatmap showing gene ontology biological processes (GOBPs) enriched by upregulated or downregulated DEGs in *Lsp1* KO T cells. The color gradient represents the enrichment score defined as –log_10_ (p value) for each GOBP. (C) Enrichment score for child GOBPs of ‘leukocyte-related biologic processes’ and ‘cell survival’. Positive value means ‘increase’ in the each GOBPs and negative value the opposite. The dotted lines indicate the cut-off levels for statistical significance. (D) Gene set enrichment analysis (GSEA) plots of ‘cell killing’ and ‘leukocyte-mediated cytotoxicity’ enriched in *Lsp1* KO T cells stimulated with media alone or anti-CD3/anti-CD28 Abs. Normalized enrichment scores (NES) and p values are presented in each plot.

We next questioned whether LSP1 regulation of tumor growth originates entirely from its effect on T cell migration. To answer this question, we unbiasedly analyzed the global transcriptome profile of *Lsp1* KO T cells (GSE75123), which was generated by our group in a previous study.^13^ As compared with WT T cells, 1256 DEGs (721 upregulated and 535 downregulated DEGs) were identified in *Lsp1* KO T cells under media or anti-CD3/anti-CD28 Abs stimulated conditions (figure 4A). We next tried to define the major cellular processes represented by the DEG (online supplemental table 1; all supplemental tables are available at https://www.cirad-catholic.com/supplementary-figures-data). Functional enrichment analysis demonstrated that the immune system process, response to stimulus, cell adhesion, localization, developmental process, cell signaling and cell killing were significantly enriched (p<0.01) by the DEGs in *Lsp1* KO T cells (figure 4B and online supplemental table 2). Among the 17 parent GOBP terms in figure 4B, we further analyzed the child GOBP terms related to leukocyte biology and then calculated their enrichment scores. As a result, ‘leukocyte-mediated migration’ and ‘leukocyte-mediated cytotoxicity’ were significantly enriched by the DEGs upregulated in *Lsp1* KO T cells, but leukocyte proliferation and survival failed to show such significance (figure 4C and online supplemental table 3). GSEA also revealed that the biological processes of ‘cell killing’ and ‘leukocyte-mediated cytotoxicity’ were increased in *Lsp1* KO T cells (figure 4D), whereas ‘negative regulation of cell killing’ was decreased (data not shown), which confirms that *Lsp1* governs the genes associated with T cell-mediated cytotoxicity. The volcano plots in online supplemental figure 7 show the 18 upregulated DEGs (78.2%) of the 23 involved in ‘leukocyte-mediated migration’ and 27 upregulated DEGs (81.8%) of the 33 involved in ‘cell killing’.

**Figure 5:**
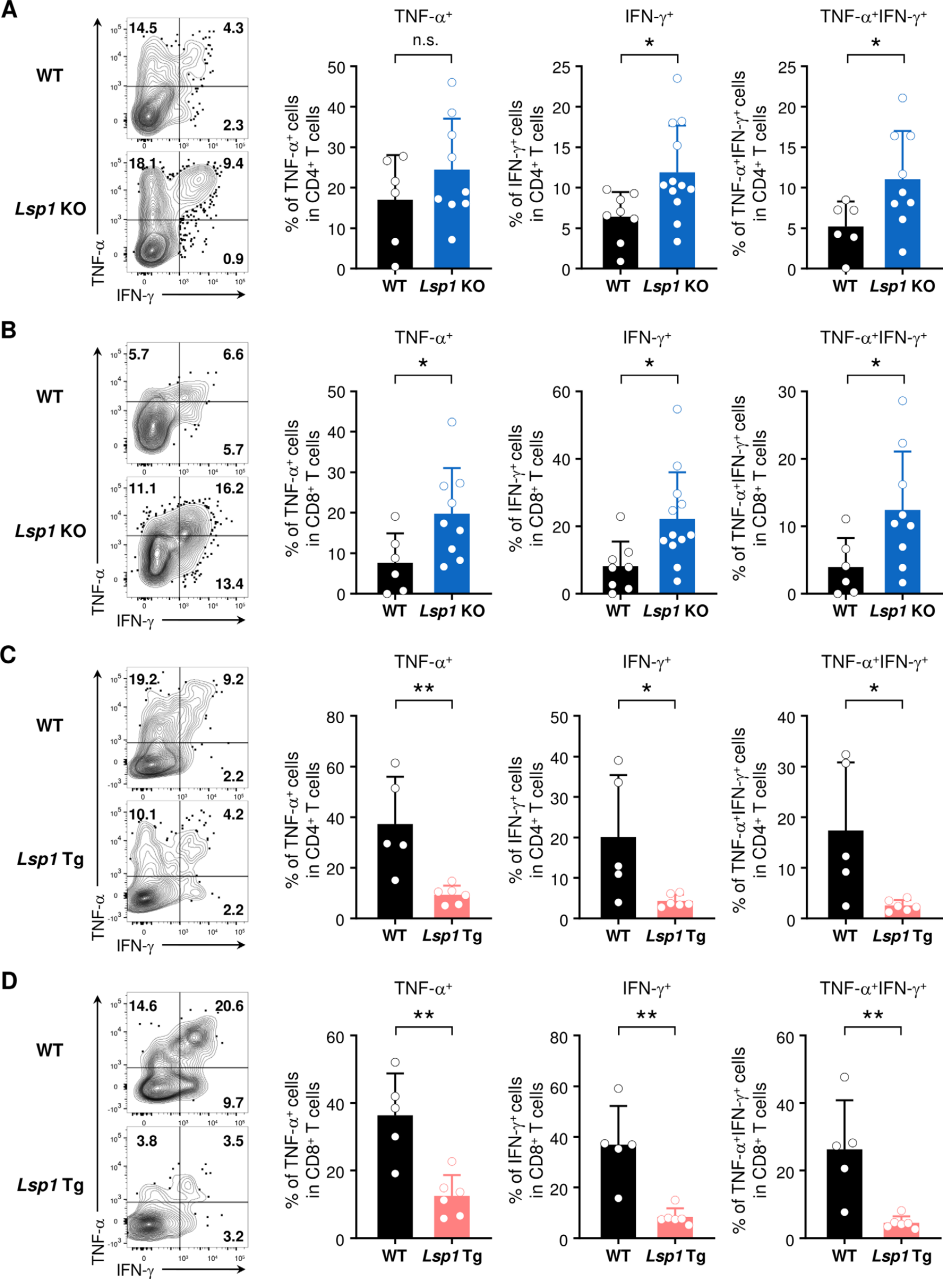
Leukocyte-specific protein 1 (LSP1) regulation of tumor necrosis factor-alpha (TNF-α) and interferon-gamma (IFN-γ) expression in tumor-infiltrating T cells. (A and B) Expression of TNF-α and IFN-γ in tumor-infiltrating T cells of *Lsp1* knockout (KO) mice. Tumor-infiltrating leukocytes (TILs) were isolated from B16 melanoma-challenged wild-type (WT) (n=6–8) and *Lsp1* KO mice (n=9–12) and then stimulated with phorbol-12-myristate-13-acetate (PMA) (50 ng/mL) and Ionomycin (500 ng/mL) in the presence of GolgiPlug for 4 hours. Expression of TNF-α and IFN-γ in CD4^+^ (A) and CD8^+^ T cells (B) was assayed by intracellular flow cytometry as described in ‘*Materials and methods*’ section. (C and D) Expression of TNF-α and IFN-γ in tumor-infiltrating T cells of *Lsp1* transgenic (Tg) (n=5) and WT mice (n=6). Intracellular cytokine staining was performed with TILs isolated from the two groups of mice, as described in (A and B). Contour plots shown on the left of (A–D) are representative data. The frequencies (%) of IFN-γ^+^ and/or TNF-α^+^ cells in CD4^+^ and CD8^+^ T cells are presented in the bar graphs as the mean±SD. P values were determined by unpaired two-tailed t-test. n.s., not significant. *P<0.05; **p<0.01.

Cytotoxicity is one of the essential steps by which tumor-infiltrating T cells suppress tumor growth.^32^ Based on our microarray data, we sought to assess if loss of *Lsp1* promotes the cytotoxic effector function of T cells. To this end, we measured the expression levels of IFN-γ and TNF-α, representative antitumor effector cytokines,^28 33^ in splenic and tumor-infiltrating T cells in WT and *Lsp1* KO mice by flow cytometry. Spleen size, the number of splenocytes and the proportion of splenic CD4^+^ and CD8^+^ T cells did not differ between tumor-bearing WT and *Lsp1* KO mice (online supplemental figure 8A–C). In the spleen, the frequencies of TNF-α^+^ and IFN-γ^+^ cells in CD4^+^ and CD8^+^ T cells were also similar between the two groups (online supplemental figure 8D, E). In the tumor, however, they were significantly higher in the infiltrated CD4^+^ and CD8^+^ T cells of *Lsp1* KO mice than in those of WT mice (figure 5A, B), suggesting that *Lsp1* deficiency increases antitumor immunity by inducing TNF-α^+^ and IFN-γ^+^ expression in tumor-infiltrating T cells.

Conversely, the frequencies of TNF-α^+^ and/or IFN-γ^+^ cells in splenic CD4^+^ and CD8^+^ cells were significantly lower in *Lsp1* Tg mice than in WT mice after tumor inoculation (online supplemental figure 9D, E). In tumor tissue, *Lsp1*-overexpressing CD4^+^ and CD8^+^ T cells also showed markedly reduced frequencies of TNF-α^+^ and/or IFN-γ^+^ cells compared with WT CD4^+^ and CD8^+^ T cells, respectively (figure 5C, D). As a control, the frequency of IL-2^+^ cells was not different in *Lsp1* Tg and WT mice. Spleen size and splenocyte numbers were also similar between the two groups of mice (online supplemental figure 9A, B). Interestingly, splenic CD8^+^ T cells of *Lsp1* Tg mice were less expanded, while splenic CD4^+^ T cells of *Lsp1* Tg mice were more expanded than those of WT mice (online supplemental figure 9C). Overall, these results suggest that *Lsp1* overexpression in T cells promotes melanoma growth through downregulation of TNF-α^+^ and IFN-γ^+^ production by CD8^+^ T cells, in addition to a marked decrease in infiltrated CD8^+^ T cells.

### Lsp1 depletion potentiates antitumor effect of anti-PD-1 antibody

**Figure 6:**
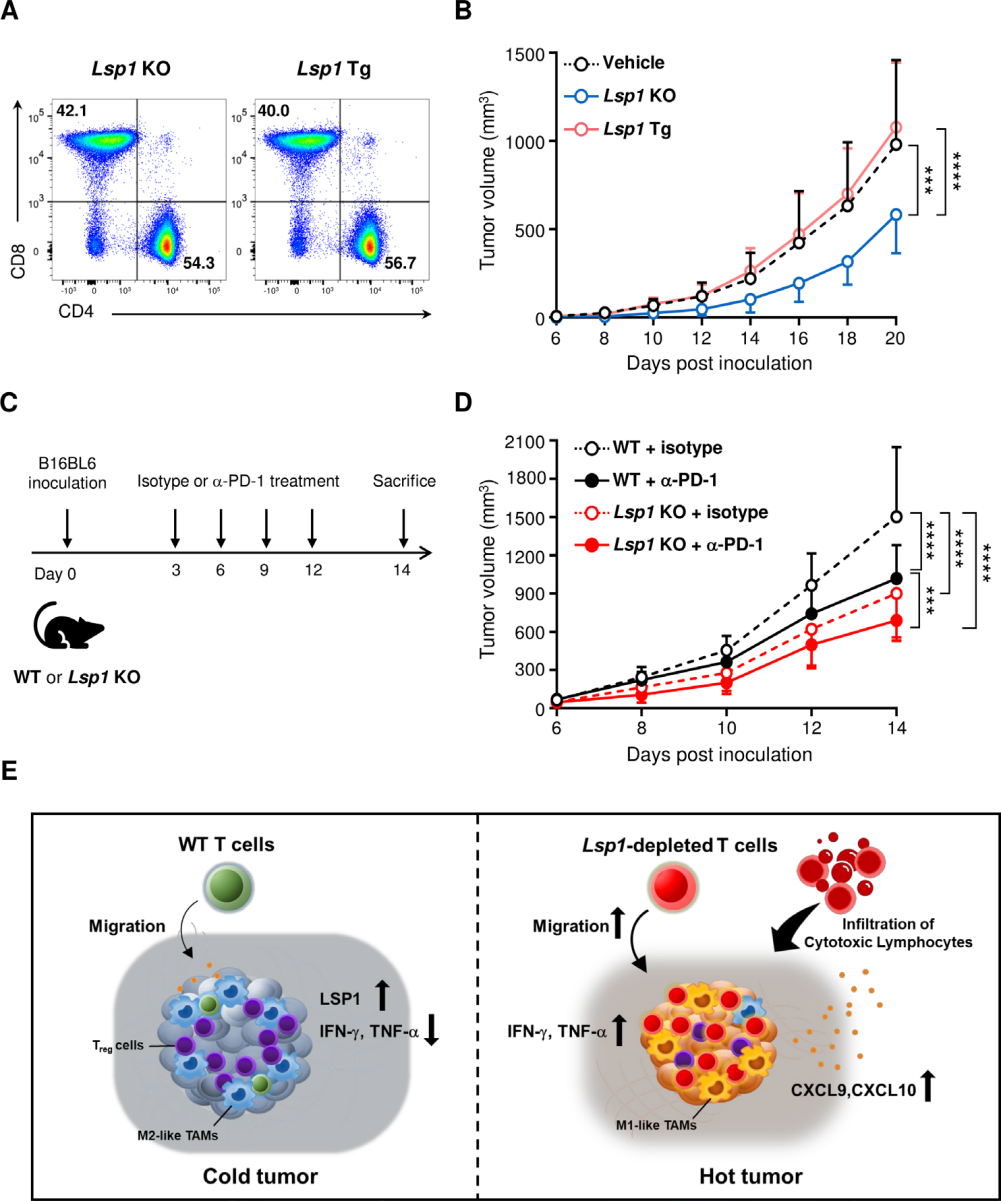
Therapeutic implications of leukocyte-specific protein 1 (LSP1) depletion in tumor progression. (A and B) Adoptive transfer of *Lsp1* knockout (KO) or *Lsp1* transgenic (Tg) T cells to *Rag1* KO mice. *Rag1* KO mice were inoculated subcutaneously with B16 melanoma on day 0. The day after tumor inoculation, 1×10^7^ splenic CD3^+^ T cells isolated from *Lsp1* KO or *Lsp1* Tg mice were intravenously injected into tumor-bearing *Rag1* KO mice. The ratio of CD4^+^/CD8^+^ T cells in the donor cells was assessed by flow cytometry prior to the injection (A). Growth curves of B16 melanoma in *Rag1* KO mice injected with vehicle alone (n=9), *Lsp1* KO T cells (n=7) and *Lsp1* Tg T cells (n=6) are presented in (B) as the mean±SD at the indicated time points. (C and D) Potentiation of antitumor effect of antiprogrammed cell death protein 1 (PD-1) antibody by *Lsp1* deficiency. *Lsp1* KO and WT mice were subcutaneously inoculated with B16 melanoma cells and then intraperitoneally injected with 10 mg/kg of either isotype control antibodies (Abs) (isotype, n=7 mice per genotype) or anti-PD-1 Abs (α-PD-1, n=10 mice per genotype) at the time points indicated in (C). Tumor growth was monitored for 14 days in the two groups of mice (D). The data shown in (B) and (D) represent the mean±SD of the two independent experiments. P values were determined by two-way analysis of variance analysis (ANOVA) with Tukey’s multiple comparison. ***p<0.001; ****p<0.0001. (E) Hypothetical model of the reconstruction of the tumor microenvironments (TME) by *Lsp1*-deficient T cells. LSP1 expression in T cells can be upregulated by stimulation of T cell receptors and interferon-gamma (IFN-γ) when exposed to the TME (figure 3F, G). Elevated LSP1 expression, in turn, may impair the antitumor effector functions of T cells, including cell migration (figure 3A), cytotoxicity (figure 4) and IFN-γ and tumor necrosis factor-alpha (TNF-α) production (figure 5). In such instances, T cell-mediated elimination of tumors is downregulated,^31^ which hampers additional release of tumor antigens and further activation of T cells, resulting in a negative feedback loop for the immunosuppressive TME.^38 39^ Conversely, if *Lsp1*-depleted T cells are generated using gene-editing technology and adoptively transferred to subjects with tumors, they seem to rapidly migrate and infiltrate into the tumor mass (figures 1 and 3A) and actively retard tumor growth (figure 6B) by producing large amounts of cytotoxic mediators, such as IFN-γ and TNF-α (figure 5). Consequently, *Lsp1*-edited T cells can induce the release of tumor antigens into the TME and augment secretion of IFN-γ-inducible chemokines, such as CXCL9 and CXCL10.^38 39 45^ These chemokines, in turn, recruit more cytotoxic T cells (figure 3A), generating a positive feedback loop for formation of an immunocompetent TME and ultimately leading to the conversion of TMEs from ‘infiltrative excluded (cold tumor)’ to ‘infiltrated-inflamed (hot tumor)’.^26 45^

On the basis of the aforementioned data, we postulated that *Lsp1*-deficient T cells more effectively suppress tumor growth due to their increased capacity for cytotoxicity as well as T cell trafficking. To address whether *Lsp1*-manipulated (eg, gene-edited) T cells have therapeutic potential and to confirm that the *Lsp1* gene in T cells is required for melanoma growth, we performed adoptive transfer experiments using *Lsp1* KO and *Lsp1* Tg T cells in *Rag1* KO mice, which are deficient in mature T and B cells.^34^ Prior to the adoptive transfer, we confirmed that *Lsp1* KO T cells and *Lsp1* Tg T cells had a similar ratio of CD4/CD8 in CD3^+^ T cells (figure 6A). As shown in figure 6B, *Lsp1*-deficient T cells more strongly repressed tumor progression in *Rag1* KO mice challenged with B16 melanoma as compared with *Lsp1*-overexpressing T cells and vehicle alone (without mature T cells), which confirms that loss of *Lsp1* in T cells specifically mediates the antitumor effect.

Anti-PD-1 blockade has been successfully used as an immunotherapy for a variety of advanced cancers, including melanoma.^35^ Finally, we investigated whether the antitumor effect of *Lsp1* deficiency can be further improved by the administration of anti-PD-1 Ab since the two approaches have different antitumor mechanisms: improvement of T cell trafficking versus blockade of inhibitory immune checkpoints, respectively. To this end, WT and *Lsp1* KO mice were subcutaneously challenged with B16 melanoma cells on day 0 followed by anti-PD-1 Ab or matched isotype control Ab on days 3, 6, 9 and 12 (figure 6C). As expected, treatment with anti-PD-1 Ab substantially reduced melanoma growth in WT mice (figure 6D). Of note, *Lsp1* KO mice treated with anti-PD-1 blockade showed a greater antitumor effect than WT mice without or with anti-PD-1 Ab (figure 6D), indicating that the extent of the antitumor effect of *Lsp1* deficiency is maintained irrespective of anti-PD-1 Ab treatment. In *Lsp1* KO mice treated with anti-PD-1 Ab, the contribution of anti-PD-1 Ab treatment and *Lsp1* deficiency to tumor suppression was 59.6% and 40.4%, respectively, as estimated by the degree of decrease in mean tumor volume 14 days after the melanoma inoculation (figure 6D).

Taken together, these observations suggest that genetic ablation of *Lsp1* in T cells is a promising strategy to boost the therapeutic efficacy of immune checkpoint inhibitors for melanoma, including anti-PD-1 Ab.

## Discussion

Evidence has emerged that the extent of intratumoral T cell infiltration in the tumor mass is one of the major factors determining the efficacy of tumor immunotherapy.^1 2 26^ In the pathology of tumor, TME constructs physical barriers and activates immunosuppressive networks so that T cell infiltration does not occur in the tumor beds.^8 31 36^ Therefore, it is essential to develop a strategy that facilitates T cell infiltration into the center of tumors. However, current anticancer immunotherapies mainly focus on reinvigorating pre-existing T cells in the tumor using immune checkpoint blockades, while strategies for accelerating T cell trafficking to the center of tumors are poorly developed.^4^

Here, we provide novel mechanisms of LSP1 regulation of tumor growth and T cell infiltration in the TME. We demonstrated first that *Lsp1* deficiency reduces the growth of B16 melanoma and enhances the infiltration of immune cells into tumor sites in mice. The effect was reproduced in an MC38 colon cancer model, indicating that LSP1 regulation of tumor progression is not limited to B16 melanoma. We also found that tumor-infiltrating T cells in WT mice were localized along the border of B16 melanoma, but rarely found in the center of a tumor. As a result, in WT mice, the distribution of immune cells, particularly T cells, showed a representative ‘infiltrated-excluded’ type of tumor, in which Ly6C^low^ F4/80^high^ TAMs prevent CD8^+^ T cell infiltration into the tumor core.^26^ By contrast, in *Lsp1* KO mice, CD4^+^ and CD8^+^ T cells were frequently found in the tumor core and their distribution represents an ‘infiltrated-inflamed’ tumor phenotype, in which cytotoxic T cells are heavily infiltrated and have potent antitumor activity.^26^

It was not clear whether the reduction of tumor growth in *Lsp1* KO mice stemmed from the increased infiltration of only CD8^+^ T cells in tumor tissue, since the frequency of CD11b^+^Ly6C^low^F4/80^high^ TAMs was also substantially decreased in the tumors of *Lsp1* KO mice. To address this issue, we created transgenic mice in which *Lsp1* is overexpressed specifically in T cells (*Lsp1* Tg mice). In sharp contrast with *Lsp1* KO mice, *Lsp1* Tg mice showed an increase in B16 melanoma growth along with a remarkable decrease in CD8^+^ T cell infiltration into the tumor sites as compared with WT mice, which indicates that *Lsp1*-overexpressing T cells directly suppress tumor growth. As expected, the frequencies of other types of immune cells did not differ between *Lsp1* Tg and WT mice, suggesting that the decrease in CD8^+^ T cells, rather than other types of TILs, in the tumor mass is one of the primary mechanisms driving the differential growth of B16 melanoma between the two groups.

Mechanistically, our data suggest how LSP1 regulates B16 melanoma growth. In vitro functional tests demonstrated that *Lsp1*-deficient CD8^+^ T cells had increased chemotactic activity via the p-Akt signaling pathway on CXCL9 and CXCL10 stimulation, the major chemokines involved in T cell trafficking towards tumor sites, whereas *Lsp1*-overexpressing CD8^+^ T cells showed the opposite response. This confirms that LSP1, an F-actin binding molecule,^10^ negatively regulates T cell migration.^13^ Intriguingly, gene expression profiling of *Lsp1* KO T cells revealed that the biological processes of ‘cell killing’ and ‘leukocyte-mediated cytotoxicity’ were significantly enriched by the DEGs. Concurrently, the expression of IFN-γ and TNF-α, major cytotoxic effector cytokines, in CD4^+^ and CD8^+^ T cells was higher in B16 melanoma of *Lsp1* KO mice than in control mice. Conversely, it was markedly decreased in tumors of *Lsp1* Tg mice, which supports our microarray data. Taken together, we believe that at least two possible mechanisms contribute to LSP1 regulation of tumor growth: 1) altered migration and infiltration of T cells into the tumor and 2) changes in the production of antitumor effector cytokines by CD8^+^ T cells.

Our data demonstrated that LSP1 directly controls the migration of CD8^+^ T cells via an intracellular signal of p-Akt on CXCL9/10 ligation. However, a recent study reported that CD4^+^ T cells influence survival and migration of CD8^+^ T cells.^37^ Thus, we wondered what the specific contribution of *Lsp1*-deficient CD4^+^ T cells is for CD8^+^ T cells migration. To address this question, WT CD8^+^ T cells were co-cultured with WT or *Lsp1* KO CD4^+^ T cells for 3 days in the presence of anti-CD3 Ab, and then their survival, proliferation and migration were examined using flow cytometry. As a result, survival and proliferation of CD8^+^ T cells did not differ between co-culture of CD8^+^ T cells with *Lsp1* KO CD4^+^ T cells and that with WT CD4^+^ T cells (online supplemental figure 10A, B). Migration of CD8^+^ T cells induced by CXCL9 and CXCL10 showed similar results (online supplemental figure 10C). Therefore, we presume that LSP1 expression level in CD4^+^ T cells has no effect on the survival, proliferation and migration of CD8^+^ T cells.

Our work may have uncovered a novel mechanism by which a tumor evades the host immune system. We found increased LSP1 expression in tumor-infiltrating T cells that are antigen-experienced in an in vivo melanoma model. In accordance with this, LSP1 levels in CD4^+^ and CD8^+^ T cells were increased by TCR stimulation in a calcineurin-dependent manner. We also demonstrated that LSP1 expression in T cells was induced by the pro-inflammatory cytokine IFN-γ. Given that the release of tumor-associated neoantigens occurs in the TME via the cancer-immunity cycle,^38^ the released tumor antigens could activate tumor-infiltrating T cells to upregulate LSP1 expression. It is plausible that IFN-γ, which is highly produced by T cells challenged with antigens, may be one of the mediators of such upregulation or may further increase LSP1 expression after activation of TCR. Taken together, elevated LSP1 expression levels induced by tumor-associated antigens or IFN-γ may hamper further migration of tumor-infiltrating T cells inside the tumor mass, resulting in establishment of the ‘infiltrated-excluded’ tumor phenotype.^26^

Insufficient T cell trafficking into the tumor sites, especially in solid tumors, reduces the therapeutic efficacy of immune checkpoint blockades.^39^ In the present study, we demonstrated that *Lsp1* KO T cells more effectively suppressed melanoma growth than *Lsp1* Tg T cells when adoptively transferred into *Rag1* KO mice, which suggests that *Lsp1*-manipulated T cells have therapeutic potential. Based on these findings, we hypothesized that an increase in T cell trafficking by *Lsp1* ablation, which can be practically achieved by gene editing using CRISPR-Cas9,^40^ would improve the efficacy of anti-PD-1 blockade. As expected, suppression of melanoma growth was more pronounced in *Lsp1* KO mice than in WT mice when treated with PD-1 Ab. Given that anti-PD-1 therapy has only a modest influence on the number of TILs in melanoma,^41 42^ the growth of melanoma might be additively regressed by the combined effects of 1) increased T cell-mediated cytotoxicity by inhibiting PD-1 and/or LSP1 and 2) enhanced T cell motility by *Lsp1* deficiency. Considering that melanoma is resistant to anti-PD-1 therapy,^43^ our data provide new evidence that adoptive cell therapy using *Lsp1*-edited T cells together with anti-PD-1 blockade might be a promising strategy for more effectively treating solid tumors, such as melanoma.

In summary, *Lsp1* KO mice showed decreased growth of B16 melanoma and increased infiltration of T cells, including CD8^+^ T cells, in the tumor mass, and these effects were completely reversed in T cell-specific *Lsp1* Tg mice. The effect of *Lsp1* deficiency was reproduced in MC38 colon cancer. LSP1 expression was elevated in tumor-infiltrating T cells and could be induced by the stimulation of T cells with TCR and IFN-γ. The CD8^+^ T cells of *Lsp1* KO mice had greater migratory capacity in response to CXCL9 and CXCL10 than those of WT mice, which was mediated through an intracellular signal of p-Akt; *Lsp1* Tg CD8^+^ T cells showed the opposite effect. Interestingly, gene expression profiling of *Lsp1* KO T cells revealed increased cytotoxicity, which seems to be associated with higher expression of IFN-γ and TNF-α in T cells. Adoptive transfer of *Lsp1* KO T cells to *Rag1* KO mice was more effective in repressing melanoma growth than transfer of *Lsp1* Tg T cells. Moreover, *Lsp1* KO mice showed a greater antitumor effect than WT mice when treated with anti-PD-1 Ab. Collectively, these results show that LSP1 regulates the growth of B16 melanoma in mice, presumably by affecting migration and infiltration of T cells into tumor sites and by modulating the production of antitumor effector cytokines by T cells. We anticipate that *Lsp1* depletion in T cells may convert immune-deficient ‘cold’ tumors to immune-sufficient ‘hot’ tumors, as depicted in figure 6E, which may serve as an effective strategy to overcome the current limitations of T cell-based immunotherapy and to improve the efficacy of immune checkpoint blockades and tumor vaccination^44^ for solid tumors.

## Conclusion

Our data provide the first evidence that LSP1 in T cells regulates the progression of melanoma. This study also demonstrates that genetic ablation of *Lsp1* in T cells improves antitumor immune response to B16 melanoma, probably by promoting T cell migration into tumor sites and by upregulating IFN-γ and TNF-α expression in T cells, which ultimately leads to the conversion of TMEs from ‘immune-resistant’ to ‘immune-susceptible’. Therefore, adoptive cell therapy using LSP1 gene-edited T cells may be an innovative strategy for treating solid tumors, including melanoma.
