# Supplementary material for: Regulation of tumor growth by leukocyte-specific protein 1 in T cells
Source: J Immunother Cancer. 2020 Oct 5;8(2):e001180. doi: 10.1136/jitc-2020-001180 (PMC7537340; doi:10.1136/jitc-2020-001180)
Supplement: Supplementary data [file jitc-2020-001180supp001.pdf]

Regulation of tumor growth by leukocyte-specific protein 1 in T cells

Riri Kwon[1,2], Bong-Ki Hong[1], Kang-Gu Lee[1,2], Eunbyeol Choi[1,2], Laurent Sabbagh[3], Chul-Soo Cho[1,4], Naeun Lee[1,*], and Wan-Uk Kim[1,2,4,*]

[1]Center for Integrative Rheumatoid Transcriptomics and Dynamics, The Catholic University of Korea, Seoul, Korea

[2]Department of Biomedicine & Health Sciences, The Catholic University of Korea, Seoul, Korea

[3]Department of Microbiology, Infectiology, and Immunology, University of Montreal, Montreal, QC, Canada

[4]Division of Rheumatology, Department of Internal Medicine, The Catholic University of Korea, Seoul, Korea

[*]To whom correspondence should be addressed:
Prof. Wan-Uk Kim, M.D., Division of Rheumatology, Department of Internal Medicine, Seoul St. Mary's Hospital, College of Medicine, The Catholic University of Korea, 222 Banpo-daero, Seocho-gu, Seoul, Korea 06591. E-mail: wan725@catholic.ac.kr, Tel: 82-2-2258-7530, Fax: 82-2-2258-7526 or Dr. Naeun Lee, Ph.D., Center for Integrative Rheumatoid Transcriptomics and Dynamics, The Catholic University of Korea, 222 Banpo-daero, Seocho-gu, Seoul, Korea 06591. E-mail: nelee2015@catholic.ac.kr, Tel: 82-2-2258-7831, Fax: 82-2-2258-7526

Running title

LSP1 regulation of melanoma growth

Keywords

Leukocyte-specific protein 1, Tumor-infiltrating lymphocytes, T cell migration, Cytotoxic T cells, B16 melanoma

Supplementary Figures

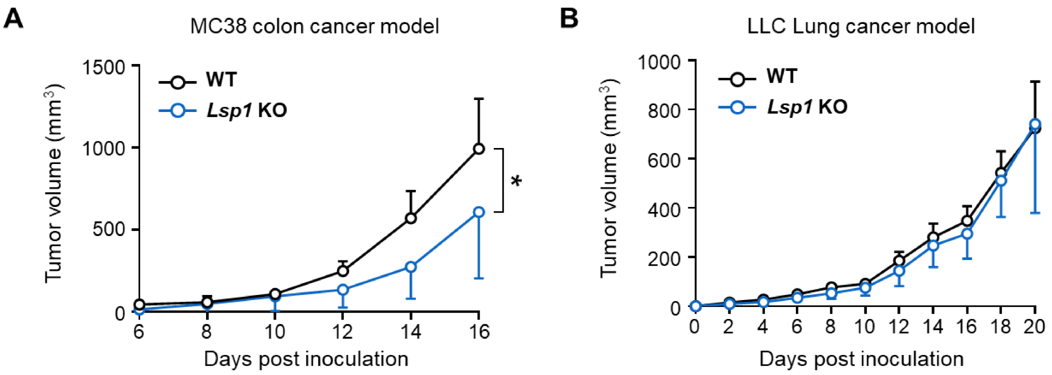

Supplementary figure 1. Growth of MC38 colon cancer and LLC lung cancer cells in WT and *Lsp1* KO mice. *Lsp1* KO (n = 5 to 8) and WT (n = 5 to 6) mice were subcutaneously inoculated with 1×10^5 MC38 cells (**A**) or 2.5×10^5 LLC cells (**B**). Tumor growth was then measured at the indicated time points.

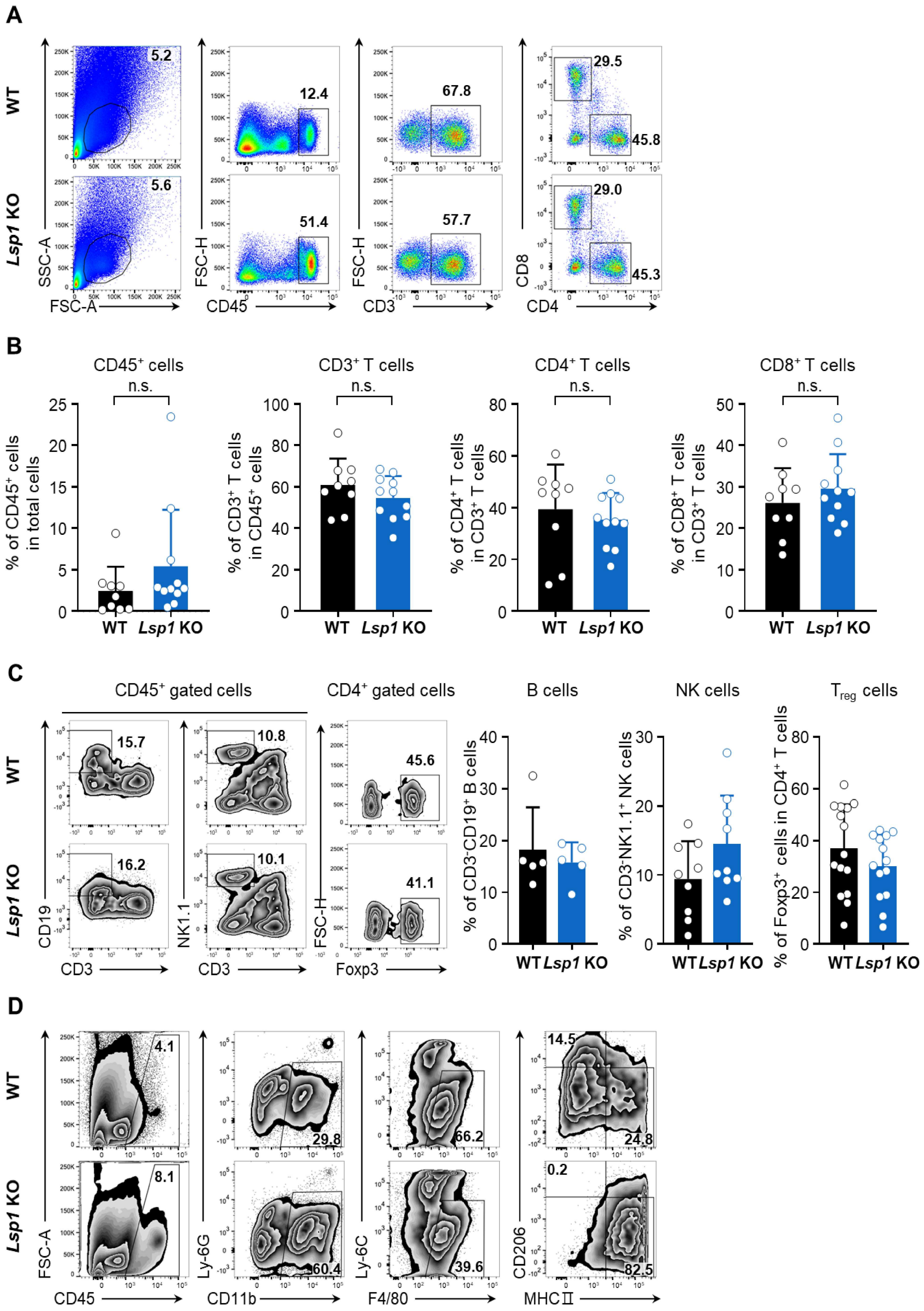
A
WT
Lsp1 KO
SSC-A
FSC-A
FSC-H
CD45
CD3
CD8
CD4
5.2
5.6
12.4
51.4
67.8
57.7
29.5
45.8
29.0
45.3
B
CD45⁺ cells
CD3⁺ T cells
CD4⁺ T cells
CD8⁺ T cells
n.s.
% of CD45⁺ cells in total cells
% of CD3⁺ T cells in CD45⁺ cells
% of CD4⁺ T cells in CD3⁺ T cells
% of CD8⁺ T cells in CD3⁺ T cells
WT
Lsp1 KO
C
CD45⁺ gated cells
CD4⁺ gated cells
B cells
NK cells
Treg cells
CD19
NK1.1
FSC-H
Foxp3
15.7
16.2
10.8
10.1
45.6
41.1
% of CD3⁻CD19⁺ B cells
% of CD3⁻NK1.1⁺ NK cells
% of Foxp3⁺ cells in CD4⁺ T cells
D
FSC-A
Ly-6G
Ly-6C
CD206
CD45
CD11b
F4/80
MHC II
4.1
8.1
29.8
60.4
66.2
39.6
14.5
24.8
0.2
82.5

38

Supplementary figure 2. Flow cytometry analysis of TILs infiltrated in B16 melanoma from WT and *Lsp1* KO mice. Mice were subcutaneously injected with 5×10^5 B16 cells. Inoculated B16 melanomas were harvested from WT and *Lsp1* KO mice when the average tumor volume in WT mice was about 700 mm^3. The immune cell populations in TILs isolated from the tumors of WT and *Lsp1* KO mice were analyzed using flow cytometry. (**A** and **B**) The frequency (%) of $CD45^+$, $CD3^+$, $CD4^+$ and $CD8^+$ T cells in TILs from WT (n = 9) and *Lsp1* KO mice (n = 11) was assessed by flow cytometry. The gating strategy for flow cytometry analysis is shown in (A). (**C**) The proportions (%) of $CD3^-CD19^+$ B cells, $CD3^-$NK 1.1^+ NK cells, and $Foxp3^+$ regulatory T cells (T_{reg} cells) in tumor-infiltrating $CD45^+$ leukocytes or $CD4^+$ T cells were compared between WT and *Lsp1* KO mice (n = 5 to 15 per group). Representative plots are shown in the left panel. (**D**) Representative zebra plots and the gating strategy for the flow cytometry analysis of $CD11b^+$ cells in $CD45^+$ leukocytes, Ly-$6C^{low}F4/80^{high}$ cells (tumor-associated macrophages; TAMs) in $CD11b^+$ cells, and $CD206^{low}MHCII^{high}$ (M1-like TAMs) or $CD206^{high}MHCII^{low}$ cells (M2-like TAMs) in Ly-$6C^{low}F4/80^{high}$ cells. Data in (**B**) and (**C**) are the mean ± SD. The circle in the bar graph indicates an individual value. *P* values were determined by unpaired two-tailed *t*-test. n.s.= not significant.

Kwon R, *et al. J Immunother Cancer* 2020; 8:e001180. doi: 10.1136/jitc-2020-001180

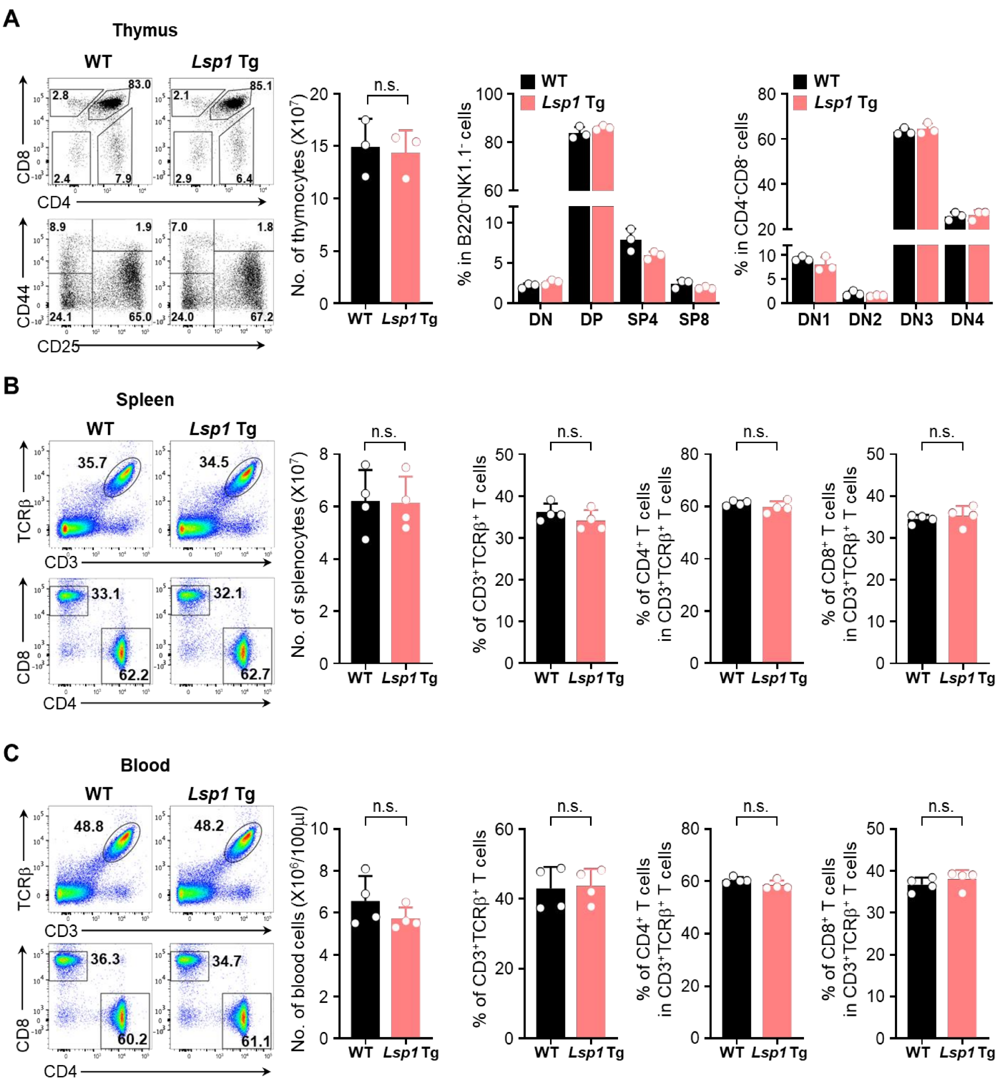

Supplementary figure 3. T cell developmental stages in thymus, blood, and spleen of WT and *Lsp1* Tg mice. (**A**) T cell developmental stages in the thymus. Thymocytes were isolated from WT and T cell-specific *Lsp1* Tg mice. Surface staining was performed for 30 minutes at 4°C with the following fluorochrome-labeled anti-mouse Abs: CD4, CD8, NK1.1, CD44, CD25, and CD45R/B220. The number of thymocytes was manually counted using a hemocytometer, and thymic developmental stages were analyzed by flow cytometry. DN,

Kwon R, *et al. J Immunother Cancer* 2020; 8:e001180. doi: 10.1136/jitc-2020-001180

double negative cells for CD4 and CD8 in B220$^-$ NK1.1$^-$ thymocytes; DP, double positive cells for CD4 and CD8 in B220$^-$ NK1.1$^-$ thymocytes; SP4, single positive cells for CD4; SP8, single positive cells for CD8; DN1, DN cells with CD44$^+$CD25$^-$; DN2, DN cells with CD44$^+$CD25$^+$; DN3, DN cells with CD44$^{-/low}$CD25$^+$; DN4, DN cells with CD44$^{-/low}$CD25$^-$. (**B** and **C**) Frequency of CD4$^+$ and CD8$^+$ T cells in blood and spleen of WT versus *Lsp1* Tg mice. Single cells were isolated from the blood and spleens from WT and *Lsp1* Tg mice. Surface staining was performed for 30 minutes at 4°C with the following fluorochrome-labeled anti-mouse Abs: CD3, TCRβ, CD4, and CD8. The number of cells was manually counted using a hemocytometer and the frequency of each subpopulation was analyzed by flow cytometry. Data in the right panels of (A) to (C) are the mean ± SD. The circle in the bar graph indicates an individual value. Representative plots and the gating strategy for flow cytometry are shown in the left panels. *P* values were determined by unpaired two-tailed *t*-test. n.s.= not significant.

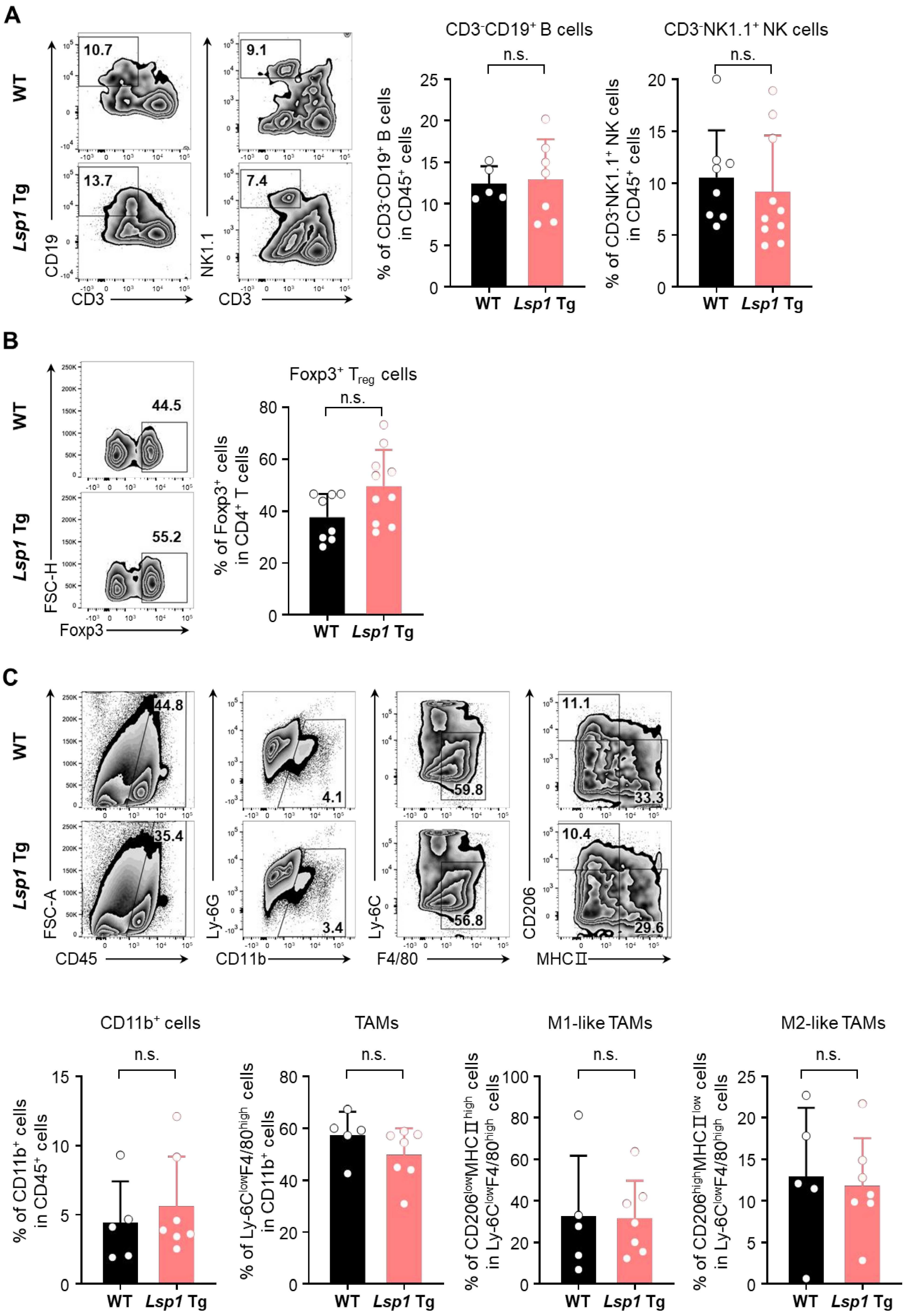

Kwon R, et al. J Immunother Cancer 2020; 8:e001180. doi: 10.1136/jitc-2020-001180

Supplementary figure 4. Immunophenotyping of TILs in tumors from WT and *Lsp1* Tg mice. Inoculated B16 melanomas were harvested from WT and T cell-specific *Lsp1* Tg mice when the average tumor volume in WT mice was about 500 mm^3. The immune cell populations in TILs isolated from the tumors of WT and *Lsp1* Tg mice were analyzed using flow cytometry. (**A**) Frequencies (%) of $CD3^-$NK 1.1^+ NK cells and $CD3^-CD19^+$ B cells in tumor-infiltrating $CD45^+$ leukocytes (n = 5 to 8 for WT mice and n = 7 to 10 for *Lsp1* Tg mice). (**B**) Frequency of $Foxp3^+$ regulatory T cells (T_{reg} cells) in $CD4^+$ T cells (n = 8 for WT mice and n = 10 for *Lsp1* Tg mice). (**C**) Frequencies of $CD11b^+$ cells in $CD45^+$ leukocytes, Ly-$6C^{low}F4/80^{high}$ cells (tumor-associated macrophages; TAMs) in $CD11b^+$ cells, and $CD206^{low}MHCII^{high}$ (M1-like TAMs) or $CD206^{high}MHCII^{low}$ cells (M2-like TAMs) in Ly-$6C^{low}F4/80^{high}$ cells (n = 5 for WT mice and n = 7 for *Lsp1* Tg mice). Representative plots and the gating strategy used are shown in the top panel. Data in the bar graphs are the mean ± SD. The circle in the bar graph indicates an individual value. *P* values were determined by unpaired two-tailed *t*-test. n.s.= not significant.

Kwon R, *et al. J Immunother Cancer* 2020; 8:e001180. doi: 10.1136/jitc-2020-001180

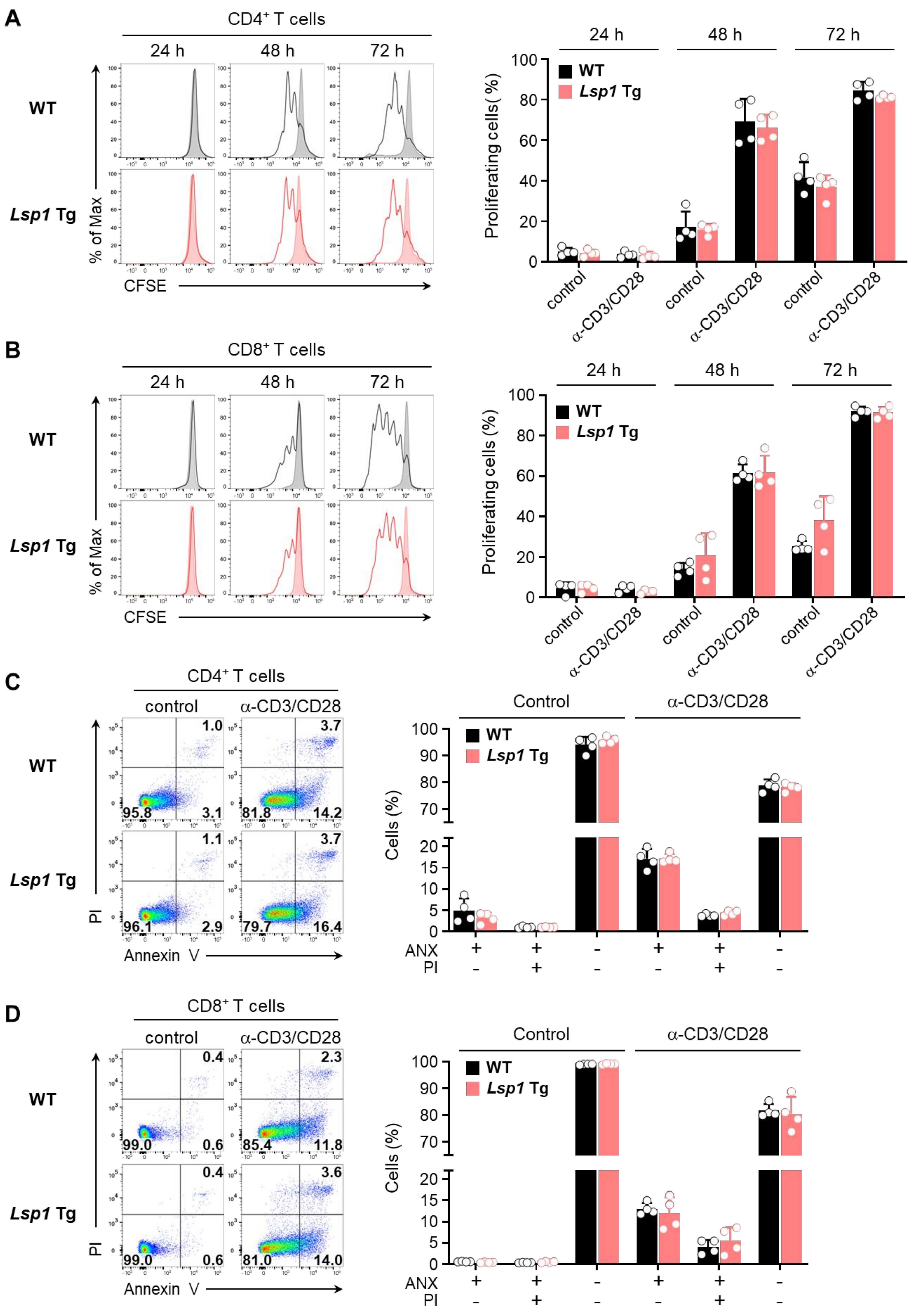
A
CD4+ T cells
24 h
48 h
72 h
WT
Lsp1 Tg
% of Max
CFSE
Proliferating cells (%)
control
α-CD3/CD28
B
CD8+ T cells
C
control
α-CD3/CD28
PI
Annexin V
Control
Cells (%)
ANX
D

89

BMJ Publishing Group Limited (BMJ) disclaims all liability and responsibility arising from any reliance placed on this supplemental material which has been supplied by the author(s)

Supplementary figure 5. No differences in T cell proliferation and survival between WT and *Lsp1* Tg mice. (**A** and **B**) CFSE-based T cell proliferation assay. Splenic $CD4^+$ (A) and $CD8^+$ T cells (B) from B16 melanoma-challenged WT and *Lsp1* Tg mice (n = 4 per genotype) were labeled with CFSE (1 μM) and then incubated for 24, 48, and 72 hours (h) in the absence or presence of anti-CD3/CD28 Abs (α-CD3/CD28, 1 μg/ml). The CFSE-diluted T cells were then analyzed by flow cytometry. (**C** and **D**) Annexin V/propidium iodide (PI) apoptosis assay of T cells. Splenic $CD4^+$ (C) and $CD8^+$ T cells (D) from B16 melanoma-challenged WT and *Lsp1* Tg mice (n = 4 per genotype) were stimulated with anti-CD3/CD28 Abs (1 μg/ml) for 48 hours and stained with FITC-annexin V (ANX) and PI. ANX^+ and/or PI^+ Cells were then analyzed by flow cytometry. Representative plots are shown in the left panels. Data in the bar graphs are the mean ± SD. The circle in the bar graph indicates an individual value. *P* values were determined by unpaired two-tailed *t*-test. n.s.= not significant.

Kwon R, *et al. J Immunother Cancer* 2020; 8:e001180. doi: 10.1136/jitc-2020-001180

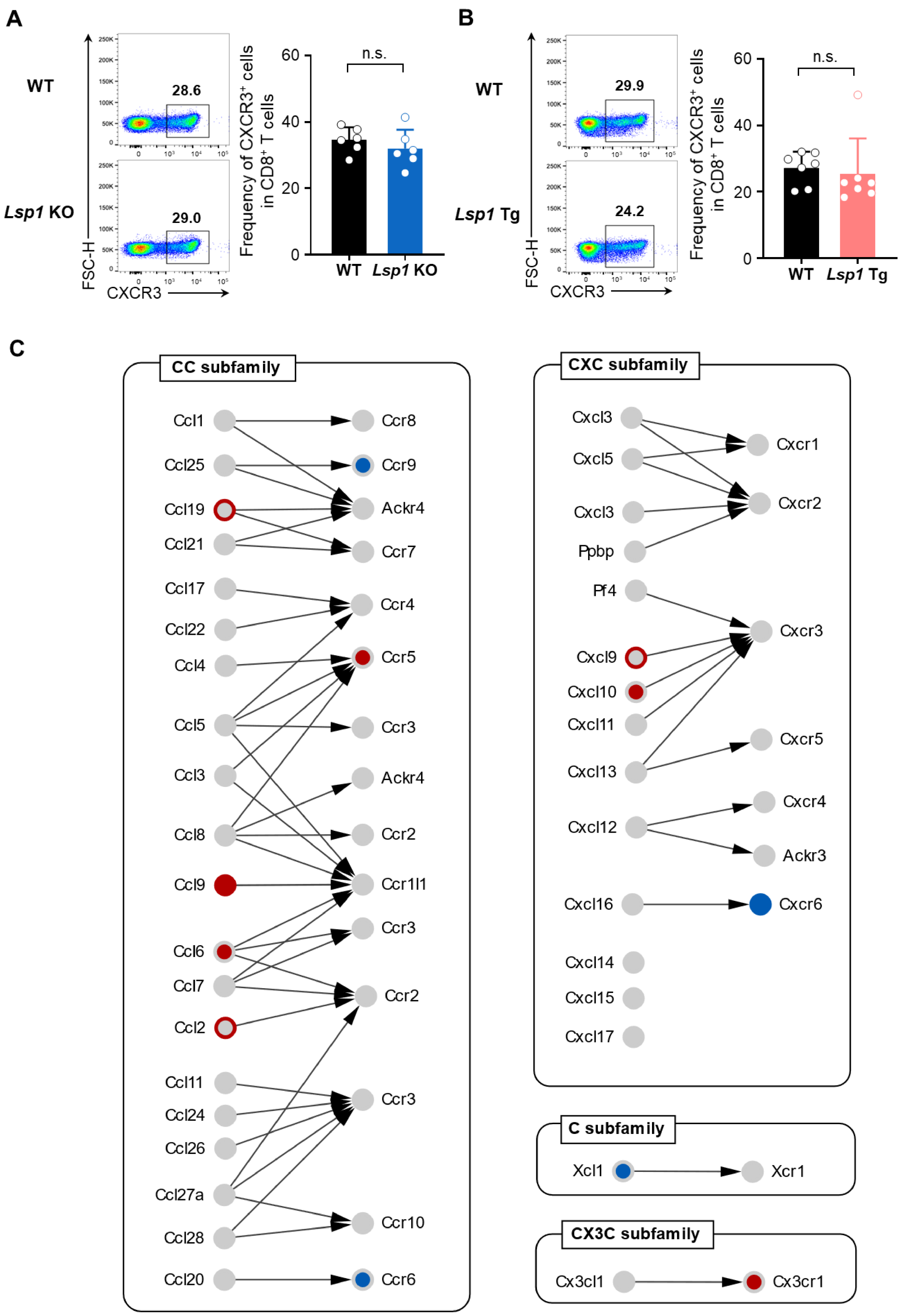
A
WT
Lsp1 KO
28.6
29.0
FSC-H
CXCR3
Frequency of CXCR3+ cells in CD8+ T cells
n.s.
WT
Lsp1 KO
B
WT
Lsp1 Tg
29.9
24.2
FSC-H
CXCR3
Frequency of CXCR3+ cells in CD8+ T cells
n.s.
WT
Lsp1 Tg
C
CC subfamily
Ccl1
Ccl25
Ccl19
Ccl21
Ccl17
Ccl22
Ccl4
Ccl5
Ccl3
Ccl8
Ccl9
Ccl6
Ccl7
Ccl2
Ccl11
Ccl24
Ccl26
Ccl27a
Ccl28
Ccl20
Ccr8
Ccr9
Ackr4
Ccr7
Ccr4
Ccr5
Ccr3
Ackr4
Ccr2
Ccr1l1
Ccr3
Ccr2
Ccr3
Ccr10
Ccr6
CXC subfamily
Cxcl3
Cxcl5
Cxcl3
Ppbp
Pf4
Cxcl9
Cxcl10
Cxcl11
Cxcl13
Cxcl12
Cxcl16
Cxcl14
Cxcl15
Cxcl17
Cxcr1
Cxcr2
Cxcr3
Cxcr5
Cxcr4
Ackr3
Cxcr6
C subfamily
Xcl1
Xcr1
CX3C subfamily
Cx3cl1
Cx3cr1

102

Supplementary figure 6. Expression of chemokine receptors in T cells of WT versus *Lsp1*-manipulated mice. (**A** and **B**) Flow cytometry analysis of CXCR3 expression, a chemokine receptor for CXCL9/10, on CD8$^+$ T cells of WT, *Lsp1* KO, and *Lsp1* Tg mice. Representative plots are shown in the left panels. Data in the bar graphs are the mean ± SD. The circle in the bar graph indicates an individual value. *P* values were determined by unpaired two-tailed *t*-test. n.s.= not significant. (**C**) KEGG pathway of the DEGs related to chemokines and chemokine receptors. The pathway reveals the chemokines and chemokine receptors upregulated (red) or downregulated (blue) in *Lsp1* KO T cells as compared to WT T cells; gray color denotes non-DEGs. The node indicates media-stimulated condition and the border anti-CD3/CD28-stimulated condition.

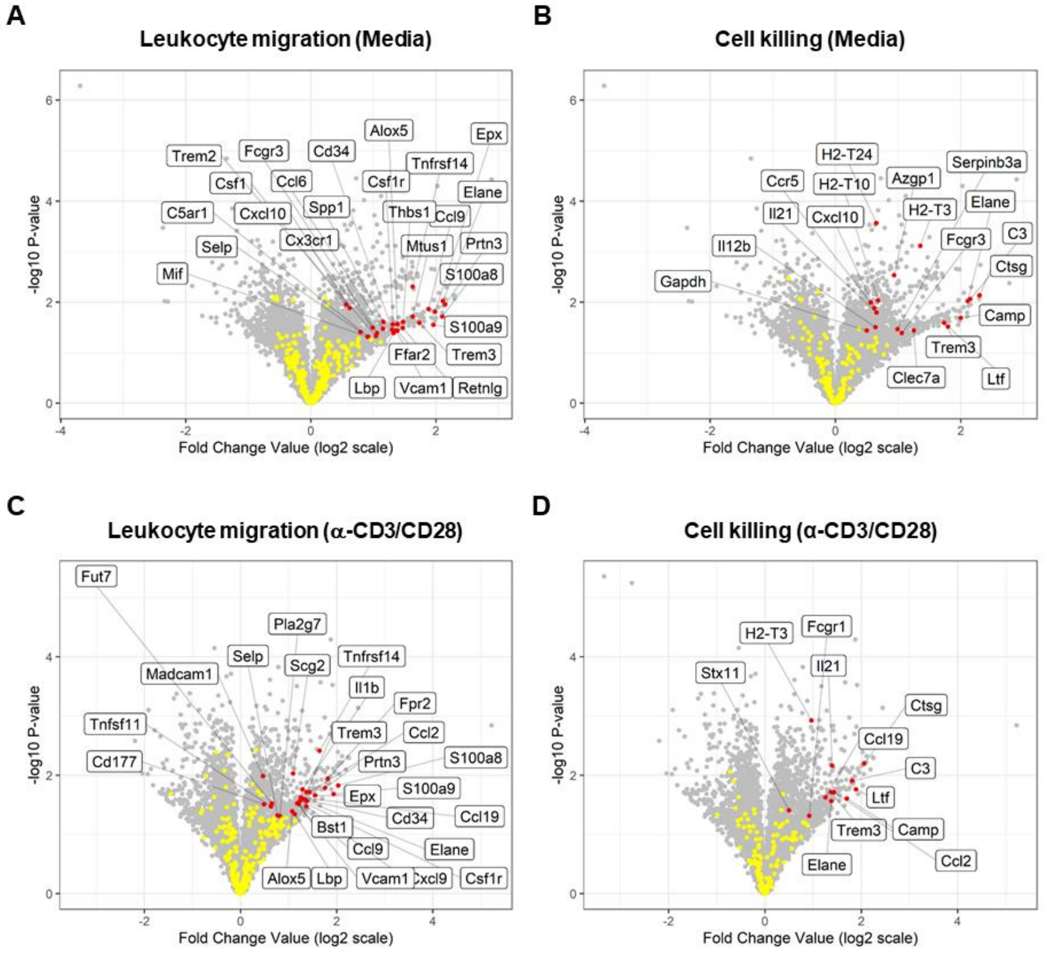

Supplementary Figure 7. Volcano plots of the DEGs involved in leukocyte migration and cytotoxicity. Each volcano plot highlights 'leukocyte migration'- and 'cell killing'-related DEGs in *Lsp1* KO T cells stimulated with media (**A** and **B**) or anti-CD3/CD28 Abs (α-CD3/CD28, **C** and **D**). The up-regulated DEGs involved in 'leukocyte migration' (GO:0050900) and 'cell killing' (GO:0001906) terms are presented with red circles and labeled by their symbols. Yellow circles denote the downregulated DEGs or non-DEGs related to the two GOBP terms. Grey circles indicate the genes representing GOBP terms other than 'leukocyte migration' and 'cell killing'.

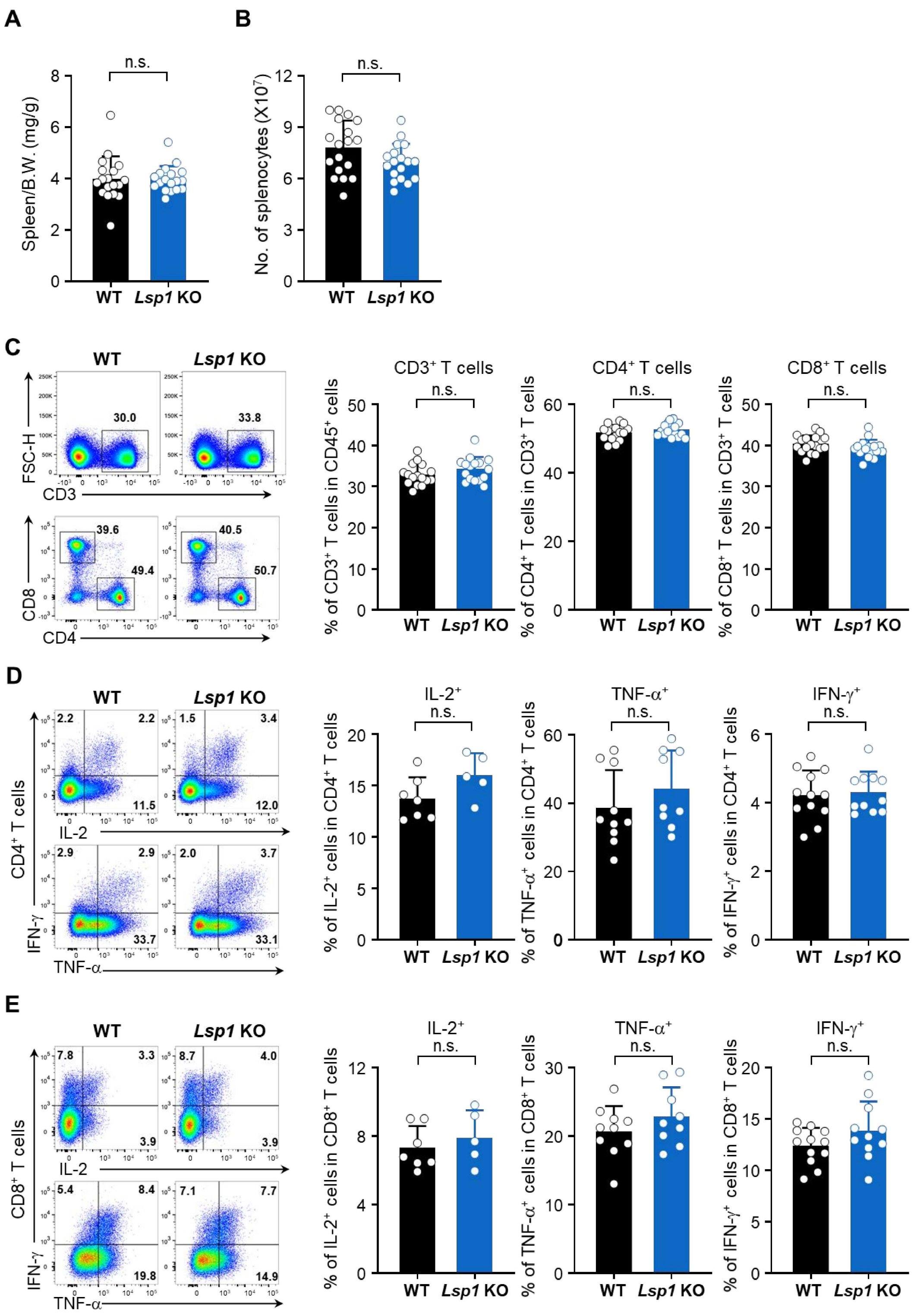
A
B
n.s.
Spleen/B.W. (mg/g)
No. of splenocytes (X10^7)
WT
Lsp1 KO
C
FSC-H
CD3
CD8
CD4
30.0
33.8
39.6
49.4
40.5
50.7
CD3+ T cells
CD4+ T cells
CD8+ T cells
% of CD3+ T cells in CD45+ cells
% of CD4+ T cells in CD3+ T cells
% of CD8+ T cells in CD3+ T cells
D
CD4+ T cells
IL-2
IFN-γ
TNF-α
IL-2+
TNF-α+
IFN-γ+
% of IL-2+ cells in CD4+ T cells
% of TNF-α+ cells in CD4+ T cells
% of IFN-γ+ cells in CD4+ T cells
E
CD8+ T cells
% of IL-2+ cells in CD8+ T cells
% of TNF-α+ cells in CD8+ T cells
% of IFN-γ+ cells in CD8+ T cells

BMJ Publishing Group Limited (BMJ) disclaims all liability and responsibility arising from any reliance placed on this supplemental material which has been supplied by the author(s)

Supplementary figure 8. Effector cytokine expression in splenic T cells of tumor-bearing WT and *Lsp1* KO mice. (A and B) As described in **figure 1B**, tumor-bearing WT (n = 18) and *Lsp1* KO mice (n = 18) were sacrificed and their spleens were harvested. The weight of each spleen was normalized relative to body weight (B.W.) (A). The number of splenocytes was counted using a hemocytometer (B). **(C)** Composition of T cells in the spleens of tumor-bearing WT and *Lsp1* KO mice as determined by flow cytometry (n = 16 per group). The frequencies of $CD3^+$ T cells in $CD45^+$ leukocytes and $CD4^+$ and $CD8^+$ T cells in $CD3^+$ T cells are shown in the representative plots (left panel) and bar graphs. (**D** and **E**) IL-2, TNF-α, and IFN-γ expression in splenic $CD4^+$ T cells (D) and $CD8^+$ T cells (E) of tumor-bearing WT (n = 7 to 12) and *Lsp1* KO mice (n = 5 to 11). The frequencies of IL-2^+, TNF-α^+, and IFN-γ^+ cells were determined by intracellular flow cytometry. T cells were stimulated with PMA (50 ng/ml) and ionomycin (500 ng/ml) in the presence of GolgiPlug for 4 hours. After incubation, the cells were harvested and surface staining was performed with fluorochrome-conjugated CD3, CD4, and CD8 Abs. The cells were fixed, permed with BD Cytofix/CytoPerm buffers, and then stained again with Abs to intracellular IL-2, TNF-α, and IFN-γ. The bar graphs represent the mean ± SD. Each symbol indicates an individual value from a different mouse. *P* values were determined by unpaired two-tailed t-test. n.s.= not significant.

Kwon R, *et al. J Immunother Cancer* 2020; 8:e001180. doi: 10.1136/jitc-2020-001180

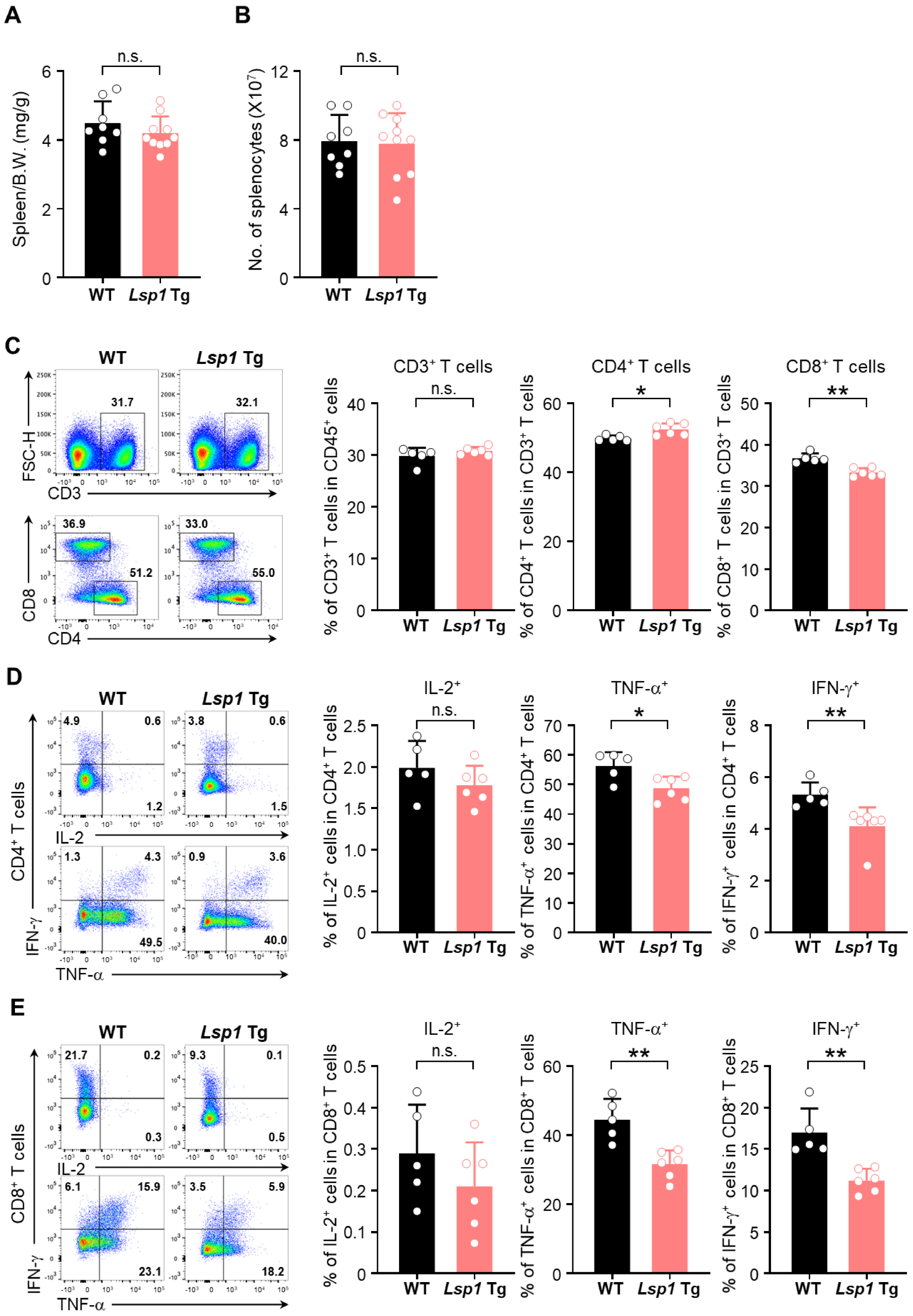

Kwon R, et al. J Immunother Cancer 2020; 8:e001180. doi: 10.1136/jitc-2020-001180

Supplementary figure 9. T cell composition and cytokine expression in splenocytes of WT and *Lsp1* Tg mice inoculated with B16 melanoma. (**A** and **B**) As described in **figure 2E**, tumor-bearing WT (n = 8) and *Lsp1* Tg (n = 10) mice were sacrificed and their spleens were harvested. The weight of each spleen was normalized relative to body weight (B.W.) (A). The number of splenocytes was counted using a hemocytometer (B). (**C**) Flow cytometry analysis of T cells in the spleens of tumor-bearing WT (n = 5) and *Lsp1* Tg mice (n = 6). The frequencies of $CD3^+$ T cells in $CD45^+$ leukocytes and $CD4^+$ and $CD8^+$ T cells in $CD3^+$ T cells are shown in the representative plots (left panel) and the bar graphs. (**D** and **E**) Frequencies of IL-2^+, TNF-α^+, and IFN-γ^+ cells in splenic $CD4^+$ T cells (D) and $CD8^+$ T cells (E) from tumor-bearing WT (n = 5) and *Lsp1* Tg mice (n = 6), as determined by intracellular flow cytometry. The bar graphs represent the mean ± SD. Each symbol indicates an individual value. *P* values were determined by unpaired two-tailed t-test. n.s.= not significant.

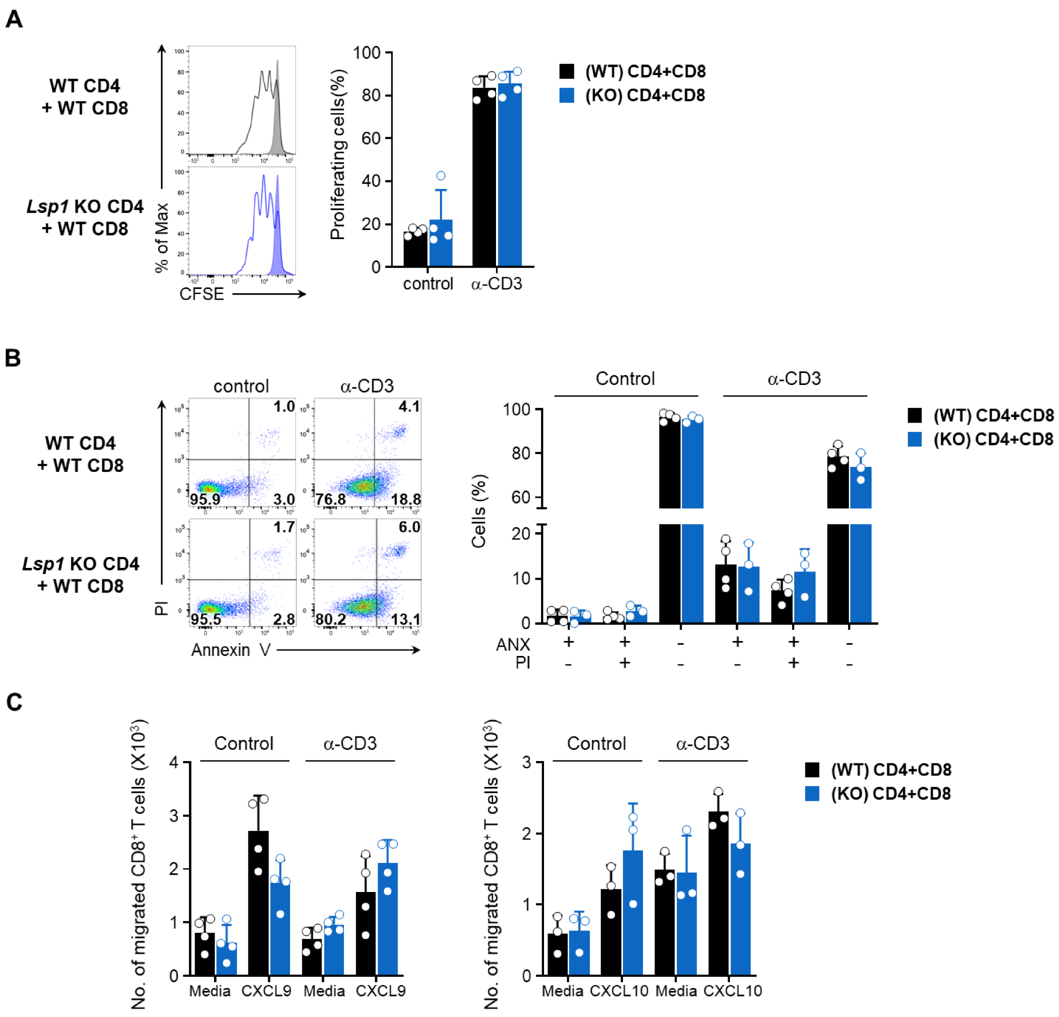

Supplementary figure 10. No effect of *Lsp1* deficiency in CD4$^+$ T cells on proliferation, survival, and migration of CD8$^+$ T cells *in vitro*. (**A**) CFSE-based T cell proliferation assay. After labeled with CFSE (1 μM), WT CD8$^+$ T cells were co-cultured with WT or *Lsp1* KO CD4$^+$ T cells in the absence or presence of anti-CD3 Ab for 3 days. CSFE-diluted CD8$^+$ T cells were then analyzed using flow cytometry. (**B**) T cell apoptosis assay. WT CD8$^+$ T cells were co-cultured with WT or *Lsp1* KO CD4$^+$ T cells in the absence or presence of anti-CD3 Ab for 3 days. The cells were harvested, stained with FITC-conjugated annexin V and PI, and then

Kwon R, *et al. J Immunother Cancer* 2020; 8:e001180. doi: 10.1136/jitc-2020-001180

subjected to flow cytometry analysis. Representative plots are shown in the left panels. **(C)** T cell migration assay. WT CD8$^+$ T cells were labeled with CFSE (1 μM) and then co-cultured with WT or *Lsp1* KO CD4$^+$ T cells in the absence or presence of anti-CD3 Ab. After 3 days, the cells were loaded in the upper chamber and allowed to migrate to the lower chamber containing CXCL9 (1000 ng/ml) or CXCL10 (1000 ng/ml). After 4 hours, CFSE-labeled CD8$^+$ T cells that migrated into the lower chamber were calculated by flow cytometry. Data in (A) to (C) are presented as the mean ± SD. The circle in the bar graph indicates an individual value. *P* values were determined by unpaired two-tailed *t*-test. n.s.= not significant.
